# Socioeconomic factors affecting outcomes in total knee and hip arthroplasty: a systematic review on healthcare disparities

**DOI:** 10.1186/s42836-022-00137-4

**Published:** 2022-10-03

**Authors:** Paul M. Alvarez, John F. McKeon, Andrew I. Spitzer, Chad A. Krueger, Matthew Pigott, Mengnai Li, Sravya P. Vajapey

**Affiliations:** 1 Department of Orthopaedics, The Ohio State University Wexner Medical Center, Columbus, USA; 2 Department of Orthopaedic Surgery, Cedars Sinai Medical Center, Los Angeles, USA; 3 Department of Orthopaedic Surgery, Rothman Orthopaedic Institute, Philadelphia, USA

**Keywords:** Healthcare disparities, Knee replacement, Hip arthroplasty, Hip replacement, Inequities, Outcomes, Insurance, Socioeconomic, Medicare, Medicaid, Hospital volume

## Abstract

**Background:**

Recent studies showed that healthcare disparities exist in use of and outcomes after total joint arthroplasty (TJA). This systematic review was designed to evaluate the currently available evidence regarding the effect socioeconomic factors, like income, insurance type, hospital volume, and geographic location, have on utilization of and outcomes after lower extremity arthroplasty.

**Methods:**

A comprehensive search of the literature was performed by querying the MEDLINE database using keywords such as, but not limited to, “disparities”, “arthroplasty”, “income”, “insurance”, “outcomes”, and “hospital volume” in all possible combinations. Any study written in English and consisting of level of evidence I-IV published over the last 20 years was considered for inclusion. Quantitative and qualitative analyses were performed on the data.

**Results:**

A total of 44 studies that met inclusion and quality criteria were included for analysis. Hospital volume is inversely correlated with complication rate after TJA. Insurance type may not be a surrogate for socioeconomic status and, instead, represent an independent prognosticator for outcomes after TJA. Patients in the lower-income brackets may have poorer access to TJA and higher readmission risk but have equivalent outcomes after TJA compared to patients in higher income brackets. Rural patients have higher utilization of TJA compared to urban patients.

**Conclusion:**

This systematic review shows that insurance type, socioeconomic status, hospital volume, and geographic location can have significant impact on patients’ access to, utilization of, and outcomes after TJA.

**Level of evidence:**

IV.

**Supplementary Information:**

The online version contains supplementary material available at 10.1186/s42836-022-00137-4.

## Background

Osteoarthritis of the hip and knee was ranked the 11^th^ highest contributor to global disability in 2010 [1]. Within the United States alone, the demand for primary THA and TKA is estimated to grow by 174% and 673%, respectively, by 2030, with economic downturns having limited impact on this rising demand [2]. However, access to beneficial orthopedic procedures may not be equal across different patient groups. Segal *et al*. showed that access to spine surgeons was significantly affected by insurance coverage—patients with private insurance were able to obtain an appointment 86.3% of the time without a primary care physician (PCP) referral while Medicaid patients could obtain an appointment 0% of the time without a PCP referral and 55% of the time with a PCP referral [3].

In addition to these observed disparities in access to orthopedic procedures, recent evidence suggests that disparities may persist in outcomes as well. Lansdown *et al*. showed that patients with Medicaid insurance had significantly lower preoperative and postoperative functional scores and had fewer follow-up visits after shoulder arthroplasty compared to patients with other insurance types [4]. These disparities in healthcare utilization and outcomes after orthopedic procedures call for a better understanding of the underlying causes so that these disparities may be mitigated. The purpose of this systematic review was to evaluate the currently available evidence regarding the effect socioeconomic factors such as income, insurance type, hospital volume, and geographic location have on utilization of and outcomes after lower extremity arthroplasty.

## Methods

### Search strategy

A search of the literature was performed by querying the MEDLINE database to identify studies that assessed healthcare disparities in patients undergoing THA or TKA. This literature search was performed in accordance with the Preferred Reporting Items for Systematic Reviews and Meta-Analyses (PRISMA) statement. All possible combinations of the following keywords were used for the search: “healthcare disparities,” “hip,” “knee,” “arthroplasty,” “joint replacement,” “THA,” “TKA,” “insurance”, “hospital volume”, “outcomes”, “social determinants”, “socioeconomic”, “payer type,” “inequities,” “inequality,” “bias,” “utilization rate,” “Medicaid,” “Medicare,” “demographic factors,” and “income.” The literature search was limited to studies published in the last twenty years from February 14, 2001, to February 14, 2021.

### Study selection

Studies meeting the following inclusion criteria were selected for the systematic review: (a) Study Level of Evidence was I, II, III, or IV as defined by Centre for Evidence-Based Medicine for therapeutic studies, (b) the study reported on results on topics within the scope of this review and (c) study had all adult participants.

**Fig. 1:**
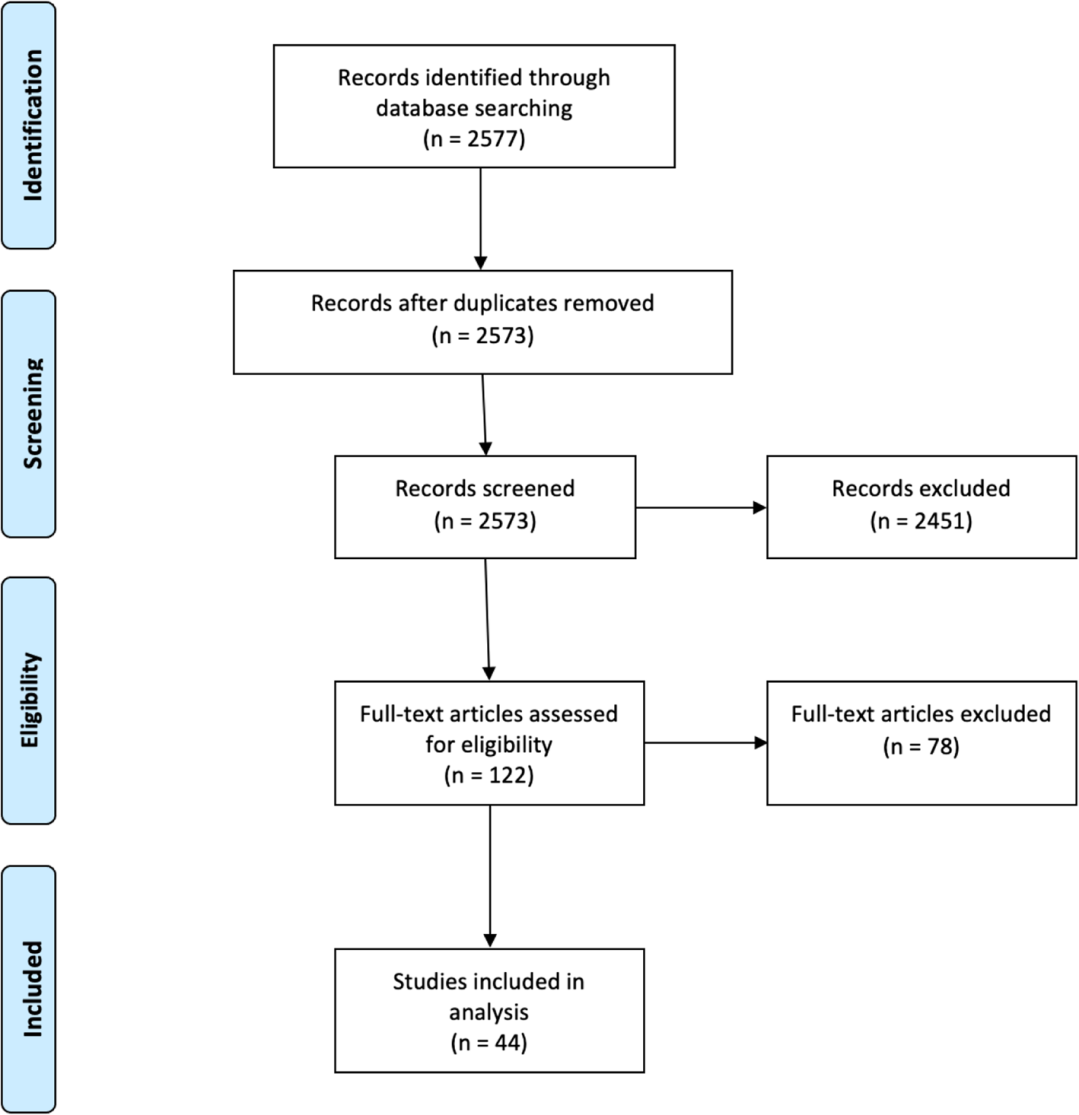
PRISMA study selection algorithm

Studies meeting the following exclusion criteria were not included in this review: (a) prior systematic reviews, (b) non-English studies, (c) case reports, expert opinions, or other studies with level V evidence, (d) basic science or biomechanical studies, (e) studies involving non-human subjects, cadavers, or pediatric patients, (f) studies involving revision surgery patients, and (g) studies conducted outside of the United States. The study selection algorithm and search results are provided in Fig. 1.

### Data extraction & analysis

The following details from each article were collected and recorded in Excel: the article title, year of publication, authors, journal published, study design, level of evidence, study question, methods, patient demographics, and pertinent results. These data were independently analyzed and synthesized.

### Quality appraisal and risk of bias assessment

The methodological quality of each study was assessed using specific criteria set forth by US Preventive Services Task Force for development of a more evidence-based approach to setting clinical practice guidelines [5]. The specific quality appraisal and risk of bias assessment criteria used to conduct this analysis are listed in the Additional file 1.

### Sources of funding

The authors received no funding for this study.

## Results

Forty-four studies met the inclusion and quality criteria. The study aims, level of evidence, methodology, and results are listed in alphabetical order of the first author in Table 1A in Additional file 1.

### Insurance type

Twenty-five studies assessed the effect of patients’ insurance type on access to and outcomes after TJA.

### Insurance type: access/utilization

Five studies assessed insurance type and its effect on access to THA evaluation. Almaguer *et al*. reported that appointments for THA evaluation were successful 99% of the time with private insurance *vs*. 72% of the time with Medicaid (*P* < 0.001) [6]. Average time to appointment was also longer for Medicaid compared to private insurance (26 days *vs*. 13 days, *P* = 0.02) [6]. Boylan *et al*. looked at use of technology assistance in TKA and THA and found that technology was more likely to be used for patients with private insurance (5.9%) compared to Medicare (4.7%, *P* < 0.001) or Medicaid (2.2%, *P* < 0.001) [7]. Hanchate *et al*. assessed the effect of socioeconomic status and insurance coverage on TKA utilization rates and reported that Medicare patients with supplemental insurance, whether it be private (OR 1.27, 95% CI 0.82–1.96) or Medicaid (OR 1.18; 95% CI 0.93–1.49), were more likely to receive a primary TKA than those without it [8]. Among middle-aged patients (age 47–64), the uninsured were much less likely to receive a primary TKA than those with private insurance (OR 0.61, 95% CI 0.40–0.92) [8]. SooHoo *et al*. reported that Medicaid insurance was an independent predictor of receiving TKA at a low-volume hospital (*P* < 0.05) [9]. Veltre *et al*. reported that privately-insured patients tend to undergo total hip replacement at higher-volume hospitals compared to Medicaid-insured or uninsured patients (46.2% *vs*. 28.7%) [10].

### Insurance type: outcomes

There were 19 studies that assessed the relationship between insurance type and outcomes after TJA.

In evaluating mortality and complications, Adelani *et al*. reported that having Medicaid insurance was associated with higher postoperative mortality (OR 1.97, 95% CI 1.49–2.59) [11]. Browne *et al*. reported that Medicaid patients had a higher prevalence of postoperative in-hospital infection (OR 1.7, 95% CI 1.3–2.1), wound dehiscence (OR 2.2, 95% CI 1.4–3.4), hematoma or seroma (OR 1.3, 95% CI 1.2–1.4) and longer length of stay but a lower risk of cardiac complications (OR 0.7, 95% CI 0.6–0.9) after TJA [12]. Maman *et al*. reported that Medicaid patients had greater odds of in-hospital mortality (OR 1.73, 95% CI 1.01–2.95, *P* < 0.05), any postoperative complications (OR 1.25, 95% CI 1.18–1.33, *P* < 0.005), extended length of stay (OR 1.09, 95% CI 1.08–1.10, *P* < 0.005) and higher total charges (OR 1.03, 95% CI 1.02–1.04, *P* < 0.005) [13]. Menendez *et al*. reported that Medicaid, but not Medicare or uninsured status was associated with higher odds of in-patient dislocation after THA (OR 1.30, 95% CI 1.02–1.65, *P* = 0.034) [14]. Plate *et al*. reported that Medicaid patients had significantly higher ASA scores (*P* < 0.001) and BMI (*P* < 0.001), with corresponding increase in procedure duration (*P* < 0.001), and prolonged LOS (*P* < 0.001) compared with other insurances, but similar to Medicare patients [15]. Veltre *et al*. reported that patients with private insurance had fewer medical complications (OR 0.80; *P* < 0.001) after THA compared to patients with Medicaid, Medicare, or no insurance [10]. Privately-insured patients also had fewer surgical complications and lower mortality after THA compared to other groups [10]. It was also reported that Medicare patients had a higher risk of mortality (relative risk [RR], 1.34; *P* < 0.001) after TKA compared to privately insured patients [16]. Xu *et al*. reported that Medicaid payer status was associated with the highest statistically significant adjusted odds of mortality (OR 2.25, 95% CI 1.01–5.01), any complications (OR, 1.26), cardiovascular complications (OR, 1.37), infectious complications (OR, 1.66) when compared with private insurance patients after THA [17].

In assessing readmission, Arroyo *et al*. reported that patients with Medicare and Medicaid insurance had higher odds of 30-day (OR 1.23, 95% CI 1.17–1.28 and OR 1.58, 95% CI 1.46–1.71 respectively) and 90-day readmission (OR 1.17, 95% CI 1.13–1.20 and OR 1.46, 95% CI 1.38–1.54 respectively) compared to private insurance holders [18]. Oronce *et al*. reported that, compared to private insurance, Medicare (OR 1.26, 95% CI 1.13–1.43), Medicaid (OR 1.86, 95% CI 1.49–2.32), and uninsured status (OR 1.31, 95% CI 1.01–1.69) were associated with increased 30-day readmission risk after THA [19]. Plate *et al*. reported that Medicare patients were significantly more likely to return to the ED (OR 3.15, 95% CI 1.88–5.27, *P* < 0.001) and be readmitted (OR 2.46, 95% CI 1.26–4.81, *P* = 0.009) compared to private or Medicaid insurance [15]. White *et al*. found that patients insured by Medicaid (OR 1.23, 95% CI 1.17–1.29) and Medicare (OR 1.58, 95% CI 1.44–1.73) had higher odds of 30-day readmission after THA compared to privately-insured patients [20]. Xu *et al*. reported that Medicaid payer status was associated with increased odds of 30-day (OR, 1.63) and 90-day readmission (OR, 1.58) after THA [17].

In assessing discharge disposition, Browne *et al*. reported that Medicaid patients had higher rates of discharge to inpatient facility after TJA (*P* < 0.01) compared to non-Medicaid insurance holders [12]. Lan *et al*. also found that non-private insurance holders had higher odds of discharge to an institution after TJA (OR 1.56, 95% CI 1.26–1.94) and having an extended length of stay [21]. Li *et al*. reported that the rate of discharge to an institution after TJA was 32.5% (95% CI 32.4%–32.7%) for Medicare-only patients, but for dual-eligible patients, the risk was similar, being at 62.3% (95% CI 61.5%–63.0%) for those with full benefits, and 61.5% (95% CI 60.7%–62.3%) for those with partial benefits [22]. Singh *et al*. reported that Medicare, Medicaid, and other insurance were associated with significantly higher odds of discharge to a rehabilitation facility, with OR of 1.77, 1.40, and 1.14, respectively, compared to private insurance [23]. Weiner *et al*. reported that Medicaid or uninsured status was associated with increased risk of non-home discharge (*P* < 0.05) [24]. In contrast, Feng *et al*. found that Medicaid status had no effect on inpatient facility discharge but was associated with longer length of stay (rate ratio 1.21, 95% CI 1.02–1.43, *P* = 0.026) [25]. Yayac *et al*. reported that Medicare Advantage patients were more likely to be discharged to a rehabilitation facility (19% *vs*. 14%, *P* < 0.0001) compared to traditional Medicare insurance patients after TJA [26].

In assessing functional outcomes, Halawi *et al*. reported that, at 1-year follow-up after TJA, Medicaid patients scored lower on PROMs (*P* < 0.01) even though net gains were comparable between Medicaid, Medicare and private insurance holders [27]. Starring *et al*. reported that Medicare patients reported significantly less ability to perform activities of daily living (78.6 *vs*. 63.2, *P* = 0.001), worse physical function (39.6 *vs*. 44.9, *P* = 0.003), and more pain interference (57.9 *vs*. 52.4, *P* = 0.018) at day 180 after TKA than commercially-insured patients [28].

### Insurance type: preoperative status

Two studies assessed preoperative status of patients with different insurance types prior to TJA. Lavernia *et al*. reported that patients with private insurance or those who were covered by Medicare had significantly better preoperative Quality of Wellbeing (QWB), SF-36, pain, and WOMAC scores relative to patients with Medicaid or those who were indigent prior to TJA (*P* < 0.01) [29]. Martin *et al*. reported that Medicaid patients had significantly worse SF-36 and WOMAC scores across all categories compared with patients with Medicare or private insurance (*P* < 0.05 for each comparison) [30]. In addition, patients with Medicaid had a higher incidence of current smoking and higher mean BMI and traveled an average of 29 to 30 miles farther for access to care (*P* < 0.05 for each comparison) [30].

**Fig. 2:**
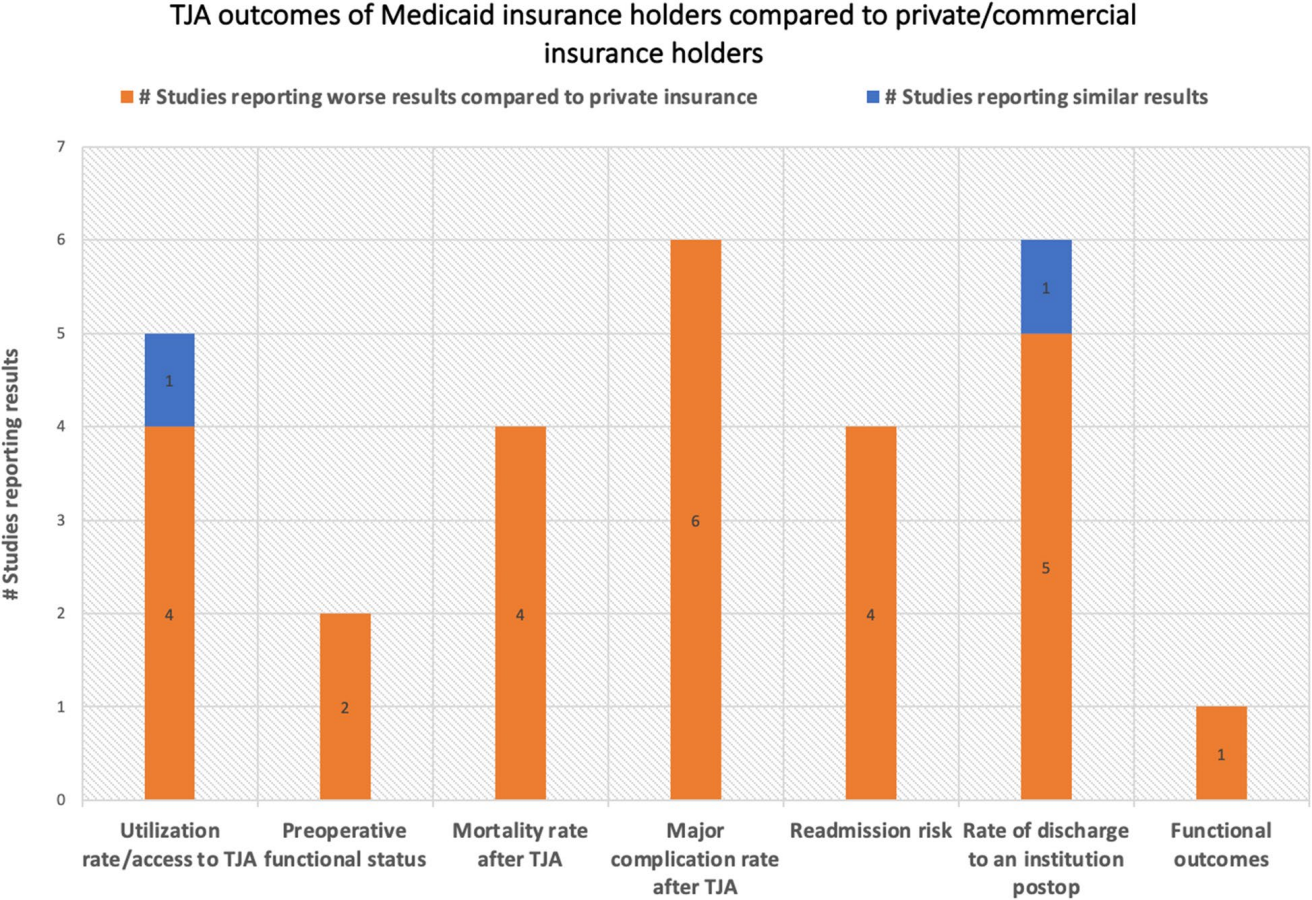
TJA outcomes of Medicaid compared to private/commercial insurance holders

**Fig. 3:**
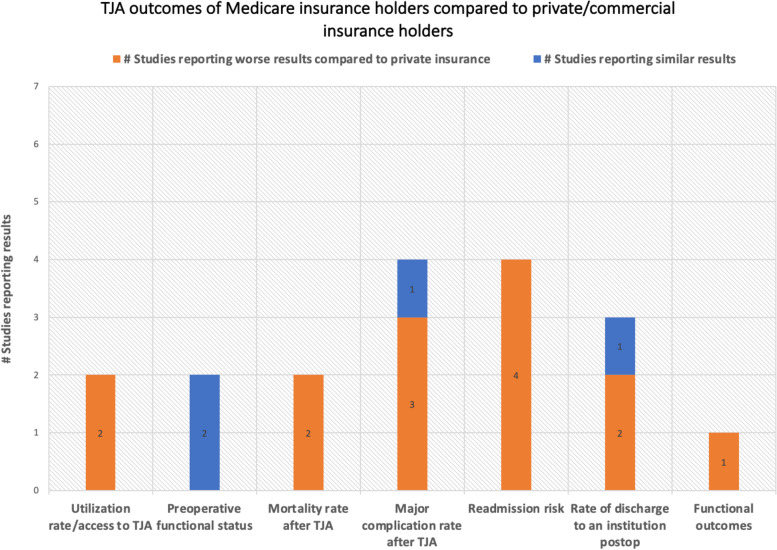
TJA outcomes of Medicare compared to private/commercial insurance holders

The healthcare disparities among the different insurance holders in terms of TJA utilization rate, surgical outcomes, and preoperative status are summarized in Figs. 2 and 3.

### Socioeconomic status

Fifteen studies assessed the impact of socioeconomic status (SES) on utilization rates of and outcomes after TJA.

### Socioeconomic status: access/utilization

There were 5 studies that assessed the impact of socioeconomic status on access to TJA. Dangelmajer *et al*. looked at utilization of hemiarthroplasty *vs*. THA for femoral neck fractures and found that there was no statistically significant difference in rates of THA for patients of different household incomes [31]. Skinner *et al*. also found little association between income and rates of TKA (OR 0.98, *P* < 0.05) but the association between TKA and income became stronger after adjusting for hospital referral region—then a 10% increase in income within a region was associated with a 1.9% increase in rate of TKA [32].

However, Hanchate *et al*. reported that those in the lowest income category (under $10K) had an estimated OR of 0.75 for receiving TKA compared to those in the highest income tier [8]. Similarly, Hawkins *et al*. reported that patients who lived in lower income areas were 5-10% (*P* < 0.001) less likely to receive a THA or TKA compared to those who resided in higher income areas [33]. SooHoo *et al*. reported that patients within the lowest income group were at increased risk of being treated at either a low-volume (relative risk ration [RRR] = 3.19, 95% CI 1.89–5.37, *P* < 0.001) or intermediate volume (RRR = 1.80, 95% CI 1.09–2.98, *P* = 0.02) hospital compared to patients within the highest income group [34].

### Socioeconomic status: outcomes

There were 10 studies that assessed the impact of socioeconomic status on outcomes after TJA.

In evaluating complication risk, Menendez *et al*. reported that lower household income ($1–$47,999: OR = 1.22, 95% CI = 1.09–1.36; $48,000–$62,999: OR = 1.16, 95% CI = 1.03–1.31; *vs*. ≥$63,000) was associated with increased odds of inpatient dislocation after primary THA [14]. However, Singh *et al*. reported that the lowest income quartile was associated with a lower likelihood of discharge to a rehabilitation/inpatient facility (HR 0.78, 95% CI 0.7– 0.79), hospital stay > 3 days (HR 0.82, 95% CI 0.80–0.83), infection (HR 0.57, 95% CI 0.50–0.65), and transfusion (HR 0.80, 95% CI 0.79–0.82) [23]. These results show that lower income does not negatively impact outcomes after total hip arthroplasty.

In evaluating revision risk, Bass *et al*. reported that community poverty was not significantly associated with TKA failure or revision [35].

In evaluating readmission risk, Arroyo *et al*. reported that, when compared to patients in the lowest median income quartile (the first quartile), patients in the higher median income quartiles all had reduced adjusted OR of 30-day and 90-day readmissions after TKA [18]. Oronce *et al*. also reported that lower socioeconomic status was associated with higher odds of 30-day readmission (OR 1.24, 95% CI 1.10–1.39) after THA [19]. White *et al*. reported that patients living in areas with higher median household income were less likely to be readmitted after TJA compared to those living in poorer areas (30-day readmission OR = 0.89, *P* < 0.05 and 90-day readmission OR = 0.91, *P* < 0.05) [20].

In evaluating discharge disposition, Inneh *et al*. reported that low and middle socioeconomic status was a significant predictor of discharge to an institution (OR 1.27, 95% CI 1.02–1.57, *P* = 0.029, and OR 1.26, 95% CI 1.10–1.44, *P* = 0.001) [36]. In contrast, Weiner *et al*. reported no significant association between household income and non-home discharge after THA [24].

In evaluating functional outcomes, Goodman *et al*. reported that higher census tract poverty level was associated with worse WOMAC pain scores at 2 years after TKA (*P* = 0.001) but this difference in pain scores did not reach minimal clinically important difference (MCID) [37]. Singh *et al*. reported that lower annual incomes of ≤ US$35, 000 (OR 0.61, 95% CI 0.40–0.94, *P* = 0.02) and > US$35, 000 to $45,000 (OR 0.68, 95% CI 0.49–0.94, *P* = 0.02) were associated with moderate to severe pain at 2 years after primary TKA but these differences disappeared by 5 years [38].

**Fig. 4:**
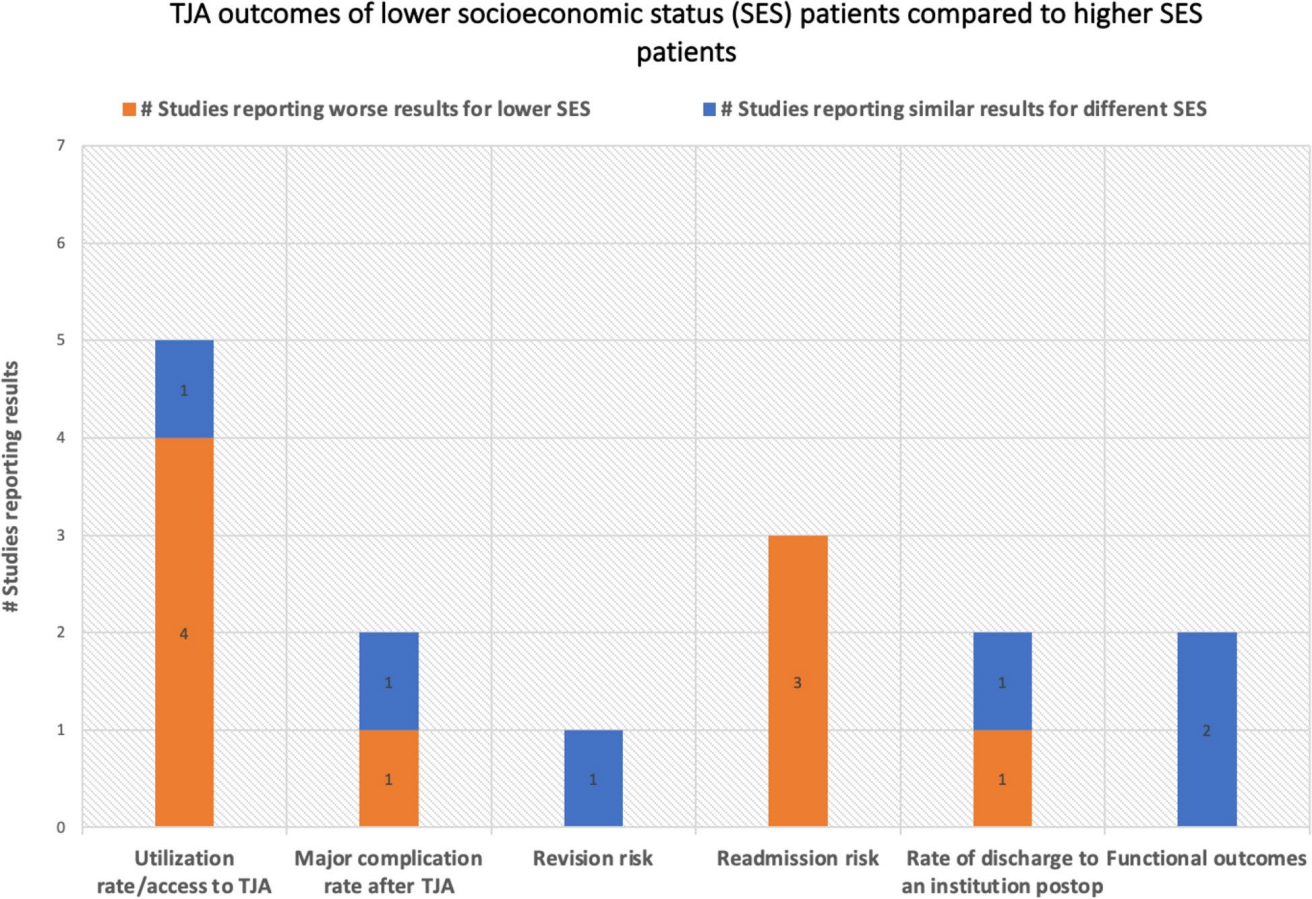
TJA outcomes of lower SES patients compared to higher SES patients

The healthcare disparities among the different socioeconomic classes in terms of TJA utilization rates and surgical outcomes are summarized in Fig. 4.

### Hospital volume

There were 9 studies that assessed the impact of hospital volume on patient outcomes after TJA.

Anis *et al*. reported that hospital volume was not found to have a significant association with revision surgery for infection or superficial infection rate when comparing high-volume to medium and low-volume hospitals [39]. In contrast, the remaining studies all showed that hospital volume was inversely correlated with complication rate after arthroplasty. Adelani *et al*. reported that the complication rate (10.2% in the lowest volume quartile to 6.7% in the highest volume quartile), readmissions (10.5% in the lowest volume quartile to 7.2% in the highest volume quartile), and ED visits (11.4% to 8.0%) after THA decreased as hospital volume increased [40]. Similarly, complications (9.1% in the lowest volume quartile to 6.8% in the highest volume quartile), readmissions (11.4% in the lowest volume quartile to 7.4% in the highest volume quartile), and ED visits (11.4–8.7%) in TKA patients decreased as hospital volume increased [40]. Doro *et al*. also reported that the highest-volume hospitals had significantly lower risk of mortality (0.16% *vs*. 0.29%, *P* < 0.001), discharge to ECF (37% *vs*. 42%, *P* < 0.001), and prolonged length of stay (14% *vs*. 23%, *P* < 0.001) after primary THA compared to low-volume hospitals [41]. Similarly, Hollenbeck *et al.* reported that procedure volume (OR 2.116, 95% CI 1.883 to 2.378) and lower patient acuity (OR 2.450, 95% CI 2.429–2.472) were independently associated with better Perfect Inpatient Care Index (PICI) scores for TJA [42]. Koltsov *et al*. reported that hospitals where less than 54 THA procedures were being performed per year had higher rate of complications (1.5-fold higher) and mortality (4-6-fold higher) after THA compared to hospitals where higher volume of THA procedures were being performed per year [43]. Laucis *et al*. also showed that very high-volume hospitals (>1000 procedures annually) had the lowest complication rates (2.745 per 100, 95% CI 2.56–2.93), and low-volume hospitals (<100 procedures annually) had the highest complication rates (3.610 per 100, 95% CI 3.58–3.64, *P* < 0.0001; OR 1.327, 95% CI 1.26–1.40) [44]. Manley *et al*. reported that TKA patients in the lowest-volume hospitals (1–25 procedures per year) had a higher risk of revision at 5 and 8 years compared with those operated on in highest-volume hospitals (>200 procedures) (OR: 1.57 and 1.52, respectively) [45].

Singh *et al*. reported that very low volume (≤25 procedures/year), low volume (26–100 procedures/year), and high volume (101–200 procedures/year) hospitals conferred a higher risk of venous thromboembolism (OR 2.0, 95% CI 0.2–16.0 *vs*. OR 3.4, 95% CI 1.4–8.0 *vs*. OR 1.1, 95% CI 0.3–3.7, respectively) and 1-year mortality (OR 2.1, 95% CI 1.2–3.6 *vs*. OR 2.0, 95% CI 1.4–2.9 *vs*. OR 1.0, 95% CI 0.7–1.5, respectively) than very high-volume (>200 procedures/year) hospitals on patients who underwent primary THA [46]. Similarly, patients who underwent primary TKA at very low-volume hospitals had significantly higher 1-year mortality rate (OR 1.6, 95% CI 1.0–2.4) compared to those who underwent TKA at very-high-volume hospitals [46]. Wilson *et al*. also found that complication rate after TKA was inversely proportional (*P* < 0.05) to hospital volume up to a point, *i.e*., complications decreased with increasing hospital volume but the rates did not differ between high volume (236 to 644 arthroplasties per year) and very high volume (≥645 arthroplasties per year) hospitals [47]. They also reported that mortality rates after TKA were significantly lower (*P* < 0.05) for hospitals with ≥645 total knee arthroplasties per year compared to those below the threshold [47].

**Fig. 5:**
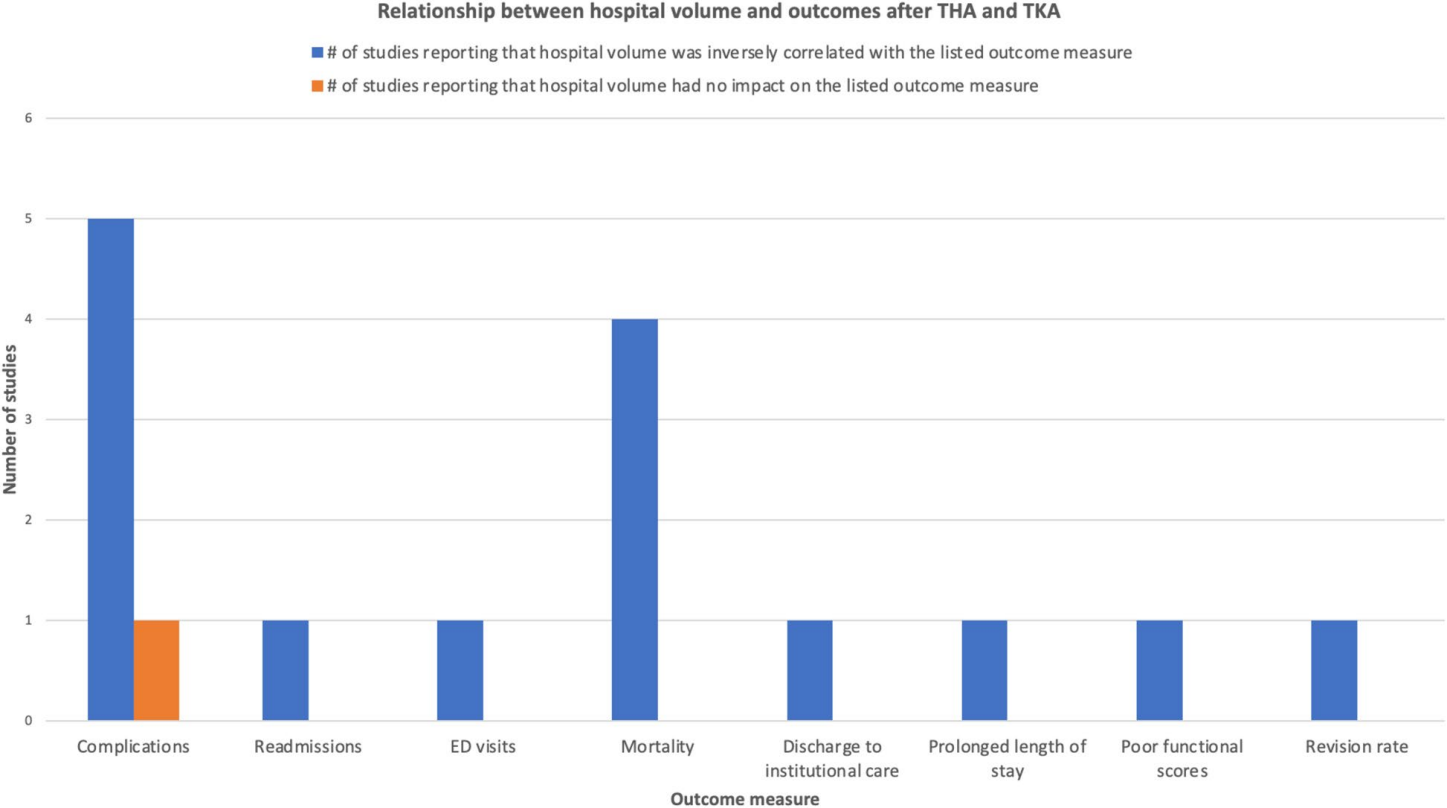
Hospital volume is inversely correlated with complication rate, readmissions, mortality, and other negative outcomes after TJA

The results of the studies on hospital volume and its impact on arthroplasty outcomes are summarized in Fig. 5.

### Geographic location

There were 2 studies that assessed the impact of geographic location of the hospital on utilization of TJA. Francis *et al*. examined utilization rates of TJA in rural *vs*. urban areas and reported that, compared to urban patients, rural patients were 27% more likely to have THA or TKA (OR 1.27, 95% CI 1.26–1.28) [48]. After adjusting for age, sex, race/ethnicity, median household income, average house value, mean poverty ratio, and state of residence, rural patients were still 14% more likely to have TJA (OR 1.14, 95% CI 1.13–1.16) [48]. Gwam *et al*. reported that the highest number of TKA procedures were being performed in the Midwest region of the United States (327 procedures per 100,000 in 2014), followed by the Northeast (211), the South (209), and the West (186) [49]. The highest number of primary TKA procedures were being performed in urban, teaching hospitals (45.3%), followed by urban, non-teaching hospitals (42.6%). Rural hospitals had the lowest percentage of primary TKA being performed every year (11.6%) [49].

## Discussion

This systematic review reveals several important findings regarding the relationship between insurance type, socioeconomic status, hospital volume, and outcomes in TJA.

The volume of the hospital can significantly affect outcomes after TJA. The results of this review show that hospital volume is correlated with outcomes after TJA—readmissions and ED visits after TJA decreased as hospital volume increased [40–47]. The highest-volume hospitals (>200 arthroplasty procedures annually) have significantly lower rates of mortality, complications, and revisions after primary TJA compared to lower-volume hospitals [40–47].

Another factor shown to affect outcomes after TJA is the patient’s insurance type. The results of this review show that Medicaid and Medicare insurance holders are more likely to have an increased risk of mortality, complications, readmissions, and discharge to institutional care after TJA compared to private/commercial insurance holders [6–8, 10–30]. Insurance type may not be a surrogate for socioeconomic status and, instead, represent an independent risk factor for outcomes.

The results of this study show that patients of lower socioeconomic status have less access to healthcare resources and higher readmission risk. Results are inconclusive in determining whether socioeconomic status has an impact on complications, functional outcomes, revisions, or discharge to institutional care after TJA [10, 14, 18–20, 23, 24, 31–33, 35–38]. Further studies are needed to delineate the relationship between socioeconomic status and outcomes after TJA.

Geographic location of patients may affect their access to TJA. The results of this review show that rural patients have higher utilization of TJA compared to urban patients, and the highest utilization of TJA is in the Midwest, followed by the South, the Northeast, and the West [48, 49].

This systematic review is subject to certain limitations. One of the weaknesses of this review is the heterogeneity of the data in the included studies. Statistical analysis of continuous variables from different studies was not possible because the included studies utilized different statistical measures (*i*.*e*., odds ratio, hazard ratio, relative risk) to report their results. Another limitation of this review is that it included studies with levels of evidence ranging from 1–4. While this ensured that our systematic review was as comprehensive as possible in capturing the effect of all social health determinants on arthroplasty outcomes, this also meant that studies with lower levels of evidence were included, which may weaken the strength of our conclusions.

Nevertheless, this review highlights important relationships between socioeconomic factors and arthroplasty outcomes. The first step in mitigating healthcare inequities is recognizing that disparities exist. Only once this first step is taken can actions items be developed to address the inequities in a patient-centered manner. This review shows that patients with Medicaid insurance, lower income status, and patients being treated at low-volume hospitals, comprise a particularly vulnerable subset. Our study showed that Medicaid holders had less access to orthopedic resources, poorer preoperative functional status, higher mortality and major complication rate, and higher readmission risk, higher rate of discharge to institutional care, and poorer functional outcomes compared to commercial insurance holders after total knee and hip arthroplasty. These findings are similar to the results of a recent study on total shoulder arthroplasty (TSA) outcomes by Singh *et al*., who showed that Medicaid insurance-holders had poorer outcomes and higher risk of complications compared to commercial insurance-holders after TSA [50]. While our study did not directly explore the reason for the impact of Medicaid insurance status on outcomes after total knee and hip arthroplasty, some possible explanations for this effect have been put forth by prior studies—less access to postoperative healthcare resources, such as physical therapy, reduced choice with respect to choosing providers, poorer preoperative functional status, and higher rates of cigarette use, obesity, and malnutrition among Medicaid insurance-holders [51].

Another key finding of our study is that patients being treated at low-volume hospitals have higher rates of complications, revision, readmission, ED visits, mortality, prolonged length of stay, discharge to institutional care, and poorer functional scores compared to patients treated at high-volume hospitals for total knee and hip arthroplasty. These findings are similar to results by a recent study on TSA outcomes by Singh *et al*., who showed that patients underwent TSA at low-volume hospital (<15 procedures annually) had higher rate of discharge to institutional care, prolonged hospital stay, postoperative fractures, blood transfusion, and revision compared to those who underwent TSA at higher-volume hospitals (>15 procedures annually) [52]. This relationship between hospital volume and arthroplasty outcomes has been consistently demonstrated across arthroplasty types (hip/knee/shoulder/revision), practice settings, time-periods, and datasets [52]. Some possible explanations that have been put forth for this observed relationship between hospital volume and arthroplasty outcomes include, but are not limited to, streamlined inpatient arthroplasty care, standardized pre-, intra- and postoperative protocols, availability of ancillary staff trained in the specialty care of arthroplasty patients, and better transition of care and discharge planning in higher-volume hospitals [52]. Other possible confounding factors are that patients referred to low-volume hospitals may differ in social support and insurance status compared to those referred to high-volume hospitals. While it creates issues with access to care if all arthroplasty patients were referred only to high-volume hospitals, it is important that patients be made aware of these findings so that they make an informed choice with regards to where they undergo arthroplasty.

## Conclusion

This systematic review shows that insurance type, socioeconomic status, hospital volume, and geographic location can have significant impact on patients’ access to, utilization of, and outcomes after TJA.

## Supplementary Information

**Additional file 1.**

## Data Availability

All data generated or analyzed during this study are included in this published article and its supplementary information files.
