# Supplementary material for: Socioeconomic factors affecting outcomes in total knee and hip arthroplasty: a systematic review on healthcare disparities
Source: Arthroplasty. 2022 Oct 3;4:36. doi: 10.1186/s42836-022-00137-4 (PMC9528115; doi:10.1186/s42836-022-00137-4)
Supplement: Supplementary file 1 — Additional file 1. [file 42836_2022_137_MOESM1_ESM.docx]

*Quality Appraisal and Risk of Bias Assessment*

A risk of bias assessment was performed for each study using the following criteria: (1) Was the selection of patients for inclusion in the study unbiased? (2) Was there systematic exclusion of any single group? (3) Was there significant attrition rate of study participants? (4) Was there a clear description of methodology and techniques in the study? (5) Was there unbiased and accurate assessment of outcomes and complications in the study? (6) Were potential confounding variables and risk factors identified and examined using acceptable statistical techniques? (7) Was the duration of follow-up reasonable for investigated outcomes? (8) Was the population included in the study described adequately? (9) Was the included participant group similar to the population at large that is affected by the condition studied? (10) Were the inclusion and exclusion criteria clearly defined? (11) Was the funding source and role of funder clearly defined in the study? (12) Were there any conflicts of interest identified or easily apparent? Only studies meeting at least ten of the twelve quality criteria above were included for analysis.

Table 1: Study design and results for all included studies

| **Author** | **Study Design (Level of Evidence)** | **Study Question** | **Demographics** | **Study Methods** | **Study Results** |
| --- | --- | --- | --- | --- | --- |
| Adelani MA, Archer KR, Song Y, et al | Retrospective cohort study (3) | Determine if there is an association between black race and adverse outcome when medical conditions were adjusted for after TJA | The patient population consisted of 585,269 patients—206,570 patients (35%) who had undergone total hip arthroplasties and 378,699 (65%) who had total knee arthroplasties. The average age was 67 years. Women comprised 61% of the patient population. Ninety-three percent of patients were white, and seven percent were Black. Hypertension was the most common medical comorbidity, affecting 54% of the total patient population. The majority of patients (88%) were treated in an urban hospital; approximately 41% were treated in teaching hospitals. | Data on 585,269 patients from the Nationwide Inpatient Samples were assessed by multivariable logistic regression analysis. Available data from 1998 through 2005 was analyzed. The outcomes of interest in this study were in-hospital postoperative complications and mortality. | Black patients were more likely to have both hypertension and diabetes than whites (P < 0.0001). Obesity was nearly twice as prevalent among Black patients compared to whites (P < 0.0001). Black patients were more likely to have Medicaid insurance coverage than whites, more likely to be treated in a teaching hospital, and more likely to be treated in hospitals with significantly lower annual case volumes (P < 0.0001 each). Multivariable logistic regression analysis demonstrated a significant association between Black race and complication after arthroplasty [OR 1.20, 95% CI 1.07–1.35]. Obesity [OR 1.23, 95% CI 1.11–1.37] and treatment in a teaching hospital [OR 1.45, 95% CI 1.23–1.71] were also associated with postoperative complication. Female patients were slightly less likely to have complications [OR 0.94, 95% CI 0.88–1.00], as were those with hypertension [OR 0.91, 95% CI 0.86–0.96]. Black race also had a significant association with death [OR 1.65, 95% CI 1.33–2.05]. Medicaid insurance [OR 1.97, 95% CI 1.49–2.59], diabetes [OR 1.37, 95% CI 1.16–1.62] and treatment in a teaching hospital [OR 1.17, 95% CI 1.02–1.35], and treatment in an urban hospital [OR 1.38, 95% CI 1.13–1.68] were also associated with postoperative mortality. Female gender [OR 0.56, 95% CI 0.50–0.63] and hypertension [OR 0.45, 95% CI 0.39–0.50] were both negatively associated with death after joint arthroplasty. This showed that when hypertension, diabetes, and obesity were accounted for, Black race remained associated with both postoperative complications and mortality. |
| Adelani MA, Keller MR, Barrack RL, et al | Retrospective cohort study (3) | The purpose of this study is to evaluate the impact of hospital volume on racial differences in outcomes following total joint arthroplasty (TJA). | A total of 340,577 patients were included in the study, 27,206 of which were African American and 23,589 of which were Hispanic. Mean age was 67.5 years old, 63.9% were female, and 20% were of low socioeconomic status. | Data was analyzed from the Healthcare Cost and Utilization Project State Inpatient Databases for patients who underwent TJA between 2006 and 20013 in New York and Florida. Complications, readmissions, and emergency department (ED) visits within 90 days of surgery were compared by hospital volume to generate relative risks. | Race/ethnicity was not associated with readmission following THA. African American race was associated with readmission following TKA (relative risk [RR] 1.16). African American race was associated with ED visits following THA (RR 1.29) and TKA (RR 1.33). Hispanic ethnicity was associated with ED visits following TKA (RR 1.15), but not THA. These associations did not change after adjusting for hospital volume. |
| Almaguer AM, Hsu AR, Pearson JM, et al. | Cross-sectional study (4) | Do patients with Medicaid seeking consultation for THA experience limits in access to evaluation for THA when compared to patients with private insurance? | 100 practices from the American Academy of Orthopedic Surgeons (AAOS) directory were called by an investigator, 5 of which were random private and 5 of which were random academic facilities from each of 5 Medicaid-expanded and 5 non-expanded states. | Practices in the 5 Medicaid-expanded and 5 non-expanded states represented different US geographic regions. Calls were made by an investigator requesting the earliest available appointment for a fictitious patient to be evaluated for a THA. Half of the calls were made with the investigator reporting private insurance of Blue Cross Blue Shield (BCBS), and half reporting Medicaid. Appointment success rate and average time to appointment were compared. Further comparisons were drawn among Medicaid-expanded vs non-expanded states, geographic regions, and private vs academic affiliation. | Appointments were successful for 99 of 100 (99%) calls made with BCBS, and 72 of 100 (72%) with Medicaid (p< .001). Success rates were significantly higher for BCBS, regardless of academic vs private affiliation. In all geographic regions, appointment success rate was significantly lower with Medicaid than with BCBS (p≤ .01). Average time to appointment was also significantly longer for Medicaid (26 days) than private (13 days) insurance (p=.020). In the Medicaid group, appointment success rate was significantly greater for academically affiliated practices compared to private practices (p= .008). |
| Anis HK, Mahmood BM, Klika AK, et al | Retrospective cohort study (3) | The purpose of this study was to evaluate the associations of hospital volume with revision surgery for infection and superficial incisional infections | There were 2640 patients (21%) in the low-volume hospital cohort, 5513 (44%) in the medium-volume cohort, and 4388 (35%) in the high-volume cohort. Several baseline characteristics were significantly different for patients treated at high-volume hospitals compared with the medium-volume and low-volume hospitals, including age, BMI, and comorbidities | A review of 12,541 primary total knee arthroplasties (TKAs) at a large integrated health system from 2014 to 2017 was conducted. Sixteen hospitals were classified as low-volume, medium-volume, or high-volume hospitals according to the mean number of TKAs/year (<250, 250-500, and >500, respectively). Thresholds were guided by percentiles and the literature on volume-outcome relationships. Medical records were reviewed for revision surgery for infection and superficial incisional infections during a mean 2-year review period. Multivariate analyses, adjusted for clinical and patient characteristics, were performed to evaluate the association between hospital volume and infection | The overall rate of revision surgery for infection was 0.7% (n = 82), and the overall rate of superficial incisional infection was 2.6% (n = 324). After accounting for potential confounders, hospital volume was not found to have a significant association with revision surgery for infection when comparing high-volume and low-volume hospitals (odds ratio, 1.615; 95% confidence interval, 0.761-3.427; P = .212) as well as when comparing high-volume and medium-volume hospitals (odds ratio, 1.464; 95% confidence interval, 0.853-2.512; P = .166). Moreover, the risk of superficial incisional infection at high-volume hospitals was similar to that at low-volume (P = .107) and medium-volume (P = .491) hospitals |
| Arroyo NS, White RS, Gaber-Baylis LK, et al | Retrospective cohort study (3) | The purpose of this study was to examine socioeconomic, racial and ethnic disparities among patients who receive elective total knee arthroplasty (TKA) within the Medicare population. | A total of 739,857 patients who underwent elective TKA and met inclusion and exclusion criteria were included within the study. The average age of the overall patient population was 67.31 years (standard deviation [SD] 10.10), with patients readmitted at 30- and 90-days being slightly older (69.23 years [SD 10.81] and 68.34 years [SD 10.85]) Females comprised 63.0% of the overall population, with 58.3% of the 30-day readmitted cohort, and 59.6% of the 90-day readmitted cohort. White patients comprised 76.4% of the overall population, with 74.7% of the 30-day readmission cohort, and 75.6% of the 90-day readmission cohort. Black patients overall represented 7.4% of the population, with 9.5% of the 30-day readmissions cohort, and 8.9% of the 90-day readmission cohort. Medicare patients represented 60.1% of the overall population, with 68.8% of the 30-day readmissions cohort, and 66.0% the 90-day readmissions cohort. Medicaid patients represented 3.1% of the overall population and 4.1% of both 30- and 90-day readmission cohorts. | The authors analyzed data from the State Inpatient Databases (SID) of the Healthcare Cost and Utilization Project (HCUP) to evaluate inpatient discharge records from California, Florida, New York, and Maryland from 2007 to 2014. Inclusion criteria included patients greater than 18 years of age who underwent TKA surgery. Patients were excluded for missing demographic data, death during the index hospitalization, insufficient follow-up time after initial hospitalization. Demographic characteristics and medical comorbidities were compared for all patients who underwent TKA during their initial inpatient hospitalization and at both 30- and 90-day readmission time points. Chi-square or Fisher exact test analysis was used to test statistical significance for categorical variables, including insurance status, readmission rates, readmission diagnoses, and demographic data. Continuous variables were compared using analysis of variance or Kruskal-Wallis tests for non-normally distributed variables. Marginal logistic regression models were used to examine the effect of markers of racial and seriocomic disparities on readmissions. | Black patients experienced higher 30-day (Odds Ratio [OR] = 1.20, 95% Confidence Interval [CI] = 1.15–1.25) and 90-day (OR = 1.08, 95% CI 1.05–1.11) readmissions when compared to white patients. Compared to patients with private insurance, Medicaid and Medicare patients had a higher likelihood of 30-day readmissions (OR = 1.17, 95% CI 1.13-1.20 and OR = 1.23, 95% CI 1.17-1.28), with similar results for 90 days (OR = 1.46, 95% CI 1.38-1.54 and OR = 1.58, 95% CI 1.46-1.71). When compared to patients in the lowest median income quartile (first quartile), patients in the higher median income quartiles all had reduced adjusted OR of readmissions. Patients treated in hospitals with higher procedure volumes (second, third, and fourth quartiles) had a lower likelihood of readmission compared to those treated at hospitals in the lowest quartile for procedure volume for white, black, Hispanic, and other race/ethnicity, Medicare- and Medicaid-insured patients continued to have higher odds of readmission. For both 30 and 90-day readmission, black patients continued to have increased odds of readmission following elective TKA when compared to white patients. Black patients with Medicare and private insurance had higher 30-day readmission rates than white patients with Medicare and private insurance; Medicaid trended positive. Diagnosis of wound infection was the most common cause of readmission for the overall study population and the number one reason for readmission among Medicaid patients. Compared to private insurance patients, Medicaid (OR = 1.22, 95% CI 1.04–1.43 and OR = 1.32, 95% CI 1.15–1.51) and Medicare (OR = 1.09, 95% CI 1.00–1.19 and OR 1.17, 95% CI 1.08–1.26) patients had increased odds of readmission for wound infection at 30 and 90 days, respectively. |
| Bass AR, Mehta B, Szymonifka J, et al | Prospective cohort study (2) | The purpose of this investigation was to determine whether racial disparities in total knee replacement (TKR) failure are explained by poverty. | A total of 4,062 patients who underwent primary unilateral TKR and who were enrolled in the Hospital for Special Surgery (HSS) Knee Replacement Registry were included in the study. The mean age was 68.4 ± 10 years, 64% of patients were female, 8% lived in census tracts with > 20% of the population under the poverty line and 9% were black. The mean follow-up was 5.3 years. | The authors included black and white New York state residents who enrolled in a single-institution TKR registry from 2008 to 2012 within the study. Patients were linked by geocoded addresses to residential census tracts. Multivariable Cox regression was used to assess predictors of TKR revision. Multivariable logistic regression was used to analyze predictors of TKR failure, defined as TKR revision in New York state within 2 years after surgery or as HSS TKR quality of life score of "not improved" or "worsened" 2 years after surgery. | A total of 3% of patients (122/4,062) required revision a median of 454 days (range 215-829) after their index procedure. Black patients were a higher risk of requiring revision (Hazard Ratio [HR] = 1.69, 95% CI 1.01-2.81). Predictors of TKR revision after multivariable analysis were noted to be younger age (HR = 0.80, 95% CI 0.74-0.88, P < 0.001), male sex, and use of a constrained prosthesis (HR = 2.31, 95% CI 1.42-3.76, P < 0.001). TKR failure occurred in 7% of patients (200/2,832) who completed 2-year surveys with risk factors for TKR failure being non-osteoarthritis TKR indication, low surgeon volume of < 50 cases a year (OR = 3.00, 95% CI 1.56-5.76, P = 0.001) and low HSS Expectations Survey score (OR = 0.84, 95% CI 0.75-0.94, P = 0.002). Black race was not a risk factor for TKR failure and community poverty was not associated with either TKR revision or failure. |
| Boylan M, Suchman K, Vigdorchik J, et al | Retrospective cohort study (3) | The purpose of this investigation was to evaluate differences in hospital and surgeon utilization of robotic and computer navigation technology when completing total joint replacement (TJR) surgery. | A total of 321,522 patients who underwent TJR surgery between 2008 and 2015 were included. 61.0% of all patients were female. 77.9% of patients were white, 8.7% were black and 1.7% were Hispanic. 37.7% of patients had private insurance, 50.8% had Medicare and 5.3% had Medicaid. | The authors analyzed the New York Statewide Planning and Research Cooperative System database to identify patients who underwent lower extremity TJR surgery between 2008 and 2015.Logistic regression models were used to determine the magnitude in difference in utilization of technology assisted arthroplasty there was among hospitals. Low volume hospitals were defined as completing 0-99 TJR surgeries a year), medium as 100-299, high as 300-799 and very high as 800 or more TJR surgeries a year. | Technology assistance was used in 5.1% of all TJR procedures included within the study. The use of technology assistance was more common for knee (7.3%) than hip (1.9%) arthroplasty (P < 0.001). The proportion of cases using technology assistance grew each year, increasing from 2.8% (knee 4.3% and hip 0.5%) in 2008 to 8.6% (knee 11.6% and hip 5.2%) in 2015 (Ptrend < 0.001). The proportion of hospitals and surgeons using robotic assistance also increased from 16.2% of hospitals and 6.2% of surgeons in 2008 to 29.2% of hospitals and 17.1% of surgeons in 2015 (Ptrend < 0.001 for both). Technology was more likely to be used for patients with private insurance (5.9%) compared with Medicare (4.7%, P < 0.001) or Medicaid (2.2%, P < 0.001), and for patients at high-volume (6.9%, P < 0.001) or very high-volume (6.1%, P < 0.001) as compared with low-volume (2.7%) hospitals. |
| Browne JA, Novicoff WM, D’Apuzzo MR | Retrospective cohort study (3) | The purpose of this study was to examine the in-hospital complications and resource utilization associated with Medicaid payer status following total joint arthroplasty (TJA). | The total number of patients analyzed was 214,217, of which 107,335 were Medicaid patients. Mean age was 56 years old. Females consisted of 69% of both Medicaid and Non-Medicaid groups. Both groups consisted of 12% Black and 11% Hispanic patients. | The Agency for Healthcare Research and Quality (AHRQ) Nationwide Inpatient Sample (NIS) database was utilized in this study. In hospital complications included those involving the central nervous system, cardiac, peripheral vascular, respiratory, gastrointestinal, and genitourinary complications; hematoma or seroma, wound dehiscence, postoperative infection, deep vein thrombosis (DVT), pulmonary embolism (PE), post-operative anemia, and mortality. Resource consumption was measured using hospital charges, length of stay, and discharge disposition. Medicaid patients were matched 1:1 with control patients who differed only in payer status. Complications, costs, and length of stay for patient with Medicaid were compared to those for patients with a different payer status. | After matching, Medicaid patients were found to have a higher prevalence of postoperative in-hospital infection (p<.01), wound dehiscence (p<.01), and hematoma or seroma (p<.01), but a lower risk of cardiac complications (p<.01). Length of hospital stay was longer, total cost was higher, and discharge to an inpatient facility was more frequent in Medicaid patients (p<.01) |
| Dangelmajer S, Yang A, Githens M, et al | Retrospective cohort study (3) | The purpose of this study was to evaluate how socioeconomic factors affect the utilization of total hip arthroplasty (THA) versus hemiarthroplasty (HA) for treatment of femoral neck fractures. | A total of 38,222 patients who underwent THA or HA for treatment of femoral neck fractures from 2009-2010. 76.8% were white, 3.6% were black, 3.4% were Hispanic, 1.3% were Asian or Pacific Islander and 1.6% were Native American. Of the patients included, 3659 underwent THA and 34,563 underwent HA. 62.8% of patients underwent surgery in a nonteaching hospital while 35.8% underwent surgery at a teaching hospital. | The authors analyzed data from the National Inpatient Sample (NIS) database to identify patients who underwent THA or HA for treatment of a femoral neck fracture. Patients were excluded if they sustained fractures at the base of the femoral neck or if the patient underwent open reduction internal fixation (ORIF). | Older patient age was associated with lower odds of receiving THA (OR = 0.944, 95% Confidence Interval [CI] 0.941-948, P < 0.0001). Asian or Pacific Islander patients had statistically lower rates of THA compared to Caucasian patients (OR = 0.507, 95% CI 0.330-0.778, P value = 0.0019). Sex and other race/ethnicities were not significantly associated with odds of receiving THA. No statistical difference was identified in the rates of THA according to median zip code income. |
| Doro C, Dimick J, Wainess R, et al | Retrospective cohort study (3) | The purpose of this study was to examine the effect of hospital volume on outcomes for primary and revision total hip arthroplasty (THA) | Non-Medicare and Medicare patients who underwent THA from the Nationwide Inpatient Sample database were analyzed. | he Nationwide Inpatient Sample database was used to identify patients for inclusion. These data include a sample of non-Medicare and Medicare patients who are unique to this study, increasing external validity compared with other studies. Outcome variables examined included in-hospital mortality and prolonged length of stay (PLOS). | Primary THA mortality was 0.16% in the highest volume quartile and 0.29% in the lowest volume quartile (P < .001). The rates of PLOS showed improved outcomes in the highest volume hospitals. Similar trends were found for revision THA, with an in-hospital mortality of 1.20% for lowest volume hospitals and 0.48% for highest volume hospitals (P < .001). |
| Feng JE, Roof MA, Adnan M, et al | Retrospective cohort study (3) | The purpose of this study is to evaluate whether point of entry and Medicaid status affect outcomes following TKA | Total number of patients included was 491, of which 174 were Medicaid insured. Medicaid patients were significantly younger (p<.01), of "other" ethnicity (p<.01), and currently smoking (p=.02). | Charts of all primary, unilateral TKA patients at a single tertiary care center between 2016 and 2018 were reviewed. TKA patients were categorized into two groups, either Hospital Ambulatory Clinic Centers patients with Medicaid insurance or private office patients with non-Medicaid insurers based on outpatient visits withing the 6-month preoperative period. Data collected included gender, age, BMI, ASA score, smoking status, operating physician. Outcomes recorded include surgical time, facility discharge, and length of stay (LOS). | After controlling for patient factors, Medicaid status had no effect on surgical time or facility discharge. Medicaid status had a significant effect on length of stay (p=.026). |
| Francis ML, Scaife SL, Zahnd WE, et al | Cross-sectional study (4) | The purpose of this investigation was to determine whether Medicare beneficiaries in rural areas were less likely to have elective total knee or hip replacement surgeries compared with their urban counterparts. | A total of 43,603,852 Medicare beneficiaries were included within the study. 87% of patients were from an urban environment and 13% were from rural environments. The average age of patients from a rural environment was 70.3 ± 12.3 and 70.8 ± 12.3 for patients from an urban environment. 90.8% of rural Medicare beneficiaries were white and 6.4% were African American (AA). 83% of urban Medicare beneficiaries were white and 10.5% were AA. Rural Medicare beneficiaries had lower median household income, average house value, and mean poverty ratio compared with urban beneficiaries. | The authors analyzed the 2005 Medicare Provider Analysis and Review File from the Centers for Medicare and Medicaid services to identify Medicare beneficiaries from both rural and urban environments who underwent primary elective total joint replacement (TJR) surgery. Exclusion criteria included patients undergoing nonelective surgery for fractures, neoplasms and revisions. The 10-point Rural-Urban Commuting Area (RUCA) codes were used to define urban and rural environments. Logistic regression was used to analyze the effect of rural or urban residency on having elective joint replacement surgery. | Compared with urban beneficiaries, rural beneficiaries were 27% more likely to have total knee or hip replacement surgeries (Odds Ratio [OR] = 1.27, 95% Confidence Interval [CI] 1.26–1.28). After adjusting for age, sex, race/ ethnicity, median household income, average house value, mean poverty ratio, and state of residence, rural beneficiaries were still 14% more likely to have total joint replacement surgeries (OR = 1.14, 95% CI 1.13–1.16). Patients from the most rural environments as defined by a RUCA of 10 were 24% more likely to undergo total knee or hip replacement surgery compared to those from the most urban environments as defined as a RUCA of 1 (OR = 1.24, 95% CI 1.23–1.26). There was, on average, a 3% increase in total joint replacement surgeries for each point along the 10-point urban to rural RUCA scale (Ptrend < 0.001). Rural patients ages 65 and older were more likely to have total joint replacement surgeries compared with urban beneficiaries (OR = 1.08, 95% CI 1.04–1.11). |
| Goodman SM, Mandl LA, Parks ML, et al | Retrospective cohort study (3) | The purpose of this study was to determine if race and socioeconomic factors at the individual level are associated with patient-reported pain and function 2 years after TKA. It also aims to determine the interaction between race and community poverty and patient reported pain and function 2 years after TKA. | The study cohort consisted of 4035 patients who underwent TKA, were enrolled in a hospital-based registry between 2007 and 2011, provided 2-year outcomes, and lived in New York, Connecticut, or New Jersey. 5% of patients who completed 2 years follow up were black. | The primary outcome was the association of race and socioeconomic factors on patient-reported outcomes after TKA. Baseline data collected on patients included age, sex, BMI, ethnicity (non-Hispanic or Hispanic), race, insurance status (Medicare, Medicaid, or other), and education (some college or above, or no college). Patient reported measures collected included preoperative Hospital for Special Surgery (HSS) Expectations score, baseline and 2-year Knee Osteoarthritis Outcomes (KOOS) pain score, and KOOS function score from which the Western Ontario and McMaster Universities OA Index (WOMAC) was derived. | Race, education, patient expectations, and baseline WOMAC scores are all associated with 2-year WOMAC pain and function, however, are not of clinical significance. White and black patients with less than 10% poverty have similar pain and function 2 years after TKA. In census tracts with greater than 40% poverty, black patients score worse than whites for WOMAC pain (p=.03) and function (p=.01). |
| Gwam C, Rosas S, Sullivan R, et al | Retrospective cohort study (3) | To assess temporal trends, primary indications, patient-level demographics, region and hospital type for all patients receiving primary total knee arthroplasty between 2009 and 2015. | There was a total of 4,283,387 TKA procedures performed from 2009 to 2015, 62.3% of which were women. Median age was 66 years old. | The National Inpatient Sample Database (NIS) was used to identify all patients who underwent a TKA between 20019 and the third quarter of 2015. Various analyses were used to assess trends, explore categorical variables, and continuous variables in the data set. Patient-level demographics included in the study were age, race, gender, health status, and median income quartile. | TKA utilization increased between 2009 and 2015 (p<.001). Primary osteoarthritis was the primary indication in 98% of cases. There was an increase in minority representation among recipients, the most being in Black patients (+2.3%, p<.001). Black TKA recipients were younger and had lower median age adjusted Charlson Comorbidity index (CCI) (p<.001). Black recipients were most likely to be of the lowest 25% of median income than any other race (p<.001). The Midwest demonstrated the greatest increase in TKAs performed per 100,000 between 2009 and 2014. TKA volume also shifted toward urban teaching hospitals (p<.001). |
| Halawi MJ, Cote MP, Savoy L, et al | Retrospective cohort study (3) | Determine the effect of payer type on PROs in total joint arthroplasty (TJA) | Medicaid patients were more likely to be smokers, live alone, have lower educational level, African American, and have nonprimary osteoarthritis as the indication for TJA | Authors' institutional joint registry was queried for patients who underwent primary, elective, and unilateral hip and knee arthroplasty. Patients were divided according to their insurance type at the time of surgery into 3 groups: Medicaid, Medicare, or commercial. The outcomes assessed were the net changes in PROs as well as absolute scores at 6 months and 1 year. Six of the most commonly used PROs were assessed: Short Form-12 physical and mental components, Western Ontario and McMaster Universities Osteoarthritis Index, Single Assessment Numerical Evaluation, University of Californian Los Angeles activity level rating, and Oxford Hip Score. Analysis of variance and covariance were used | They evaluated 756 procedures (273 Medicaid, 270 Medicare, and 213 commercial insurance). Medicaid patients had significantly lower mean baseline scores across all PROs compared to either Medicare or commercial insurance patients. At 1-year follow-up, the net mean outcome gains were comparable among the 3 payer types (P > .05), but Medicaid patients continued to score lower while Medicare and commercial insurance patients continued to score higher (P < .01). When adjusting for all baseline differences among Medicaid patients, the negative effects of payer type resolved except for Oxford Hip Score which remained lower in the Medicaid group (P = .006) |
| Hanchate AD, Zhang Y, Felson DT, et al | Retrospective cohort study (3) | The purpose of this study is to estimate national total knee arthroplasty (TKA) rates by economic factors, and the extent to which differences in insurance coverage, income, and assets contribute to racial and ethnic disparities in TKA use. | The study included 55,469 person-year observations from 18,439 patients, 57% of which were women, and 14% were non-Hispanic Black. 663 patients had a TKA between 1998 and 2004. | Longitudinal Health and Retirement Study data was used for analysis. The primary outcome is a binary indicator of whether a patient received a primary TKA in the survey period. Access to health insurance was categorized in 7 groups, income was summed from all sources, and key demographics collected include age, gender, and race. | After adjusting for economic factors, racial/ethnic differences in TKA rates for women did not occur. Compared to white men, there remained a large deficit for black men (p<.05). Among patients between ages 47-64, compared with the privately insured, those who were uninsured were less likely (95% CI 0.4-0.92) and those with Medicaid were more likely (95% CI 1.03-2.26) to receive their first TKA. |
| Hawkins K, Escoto KH, Ozminkowski RJ, et al | Retrospective cohort study (3) | The purpose of this study was to determine if disparities exist within osteoarthritis patients with AARP-branded Medicare supplement plan coverage provided by UnitedHealthcare. | 2.2 million patients were eligible for the study, 529,652 (24%) of which had osteoarthritis (OA). Of these, 6.1% received a total hip arthroplasty (THA) or total knee arthroplasty (TKA). 70% of OA patients were female, and 77% were between ages 65-84 years old. | Patients were selected into the study if they had one or more health care claims with a primary diagnosis of OA at any time from 7/1/2006 to 6/30/2007. Various analyses were used to describe patients and their utilization of hip or knee replacement surgery, and eliminate confounding effects of variables such as demographics, socioeconomics, and health status. | Males were more likely than females to receive a replacement surgery by 6% (p<.001). Patients in minority (p<.001) or lower income neighborhoods (p<.001) were less likely to receive a THA or TKA. The largest disparities existed by residential location and comorbid condition. |
| Hollenbeck B, Hoffman MA, Tromanhauser SG | Retrospective cohort study (3) | Determine how hospital volume and other factors affect quality for patients undergoing total hip and knee arthroplasty. | From 2001 to 2011, the NIS database reported 1,651,354 total hip or total knee arthroplasties. | Using the NIS of the Healthcare Cost and Utilization Project (HCUP) of the Agency for Healthcare Research and Quality (AHRQ), authors conducted a retrospective study of all total hip and total knee arthroplasties performed from 2001 to 2011. They identified all procedure and outcome variables using the International Classification of Diseases, Ninth Revision (ICD-9) billing codes. Patients were grouped into quartiles based on the corresponding hospital's procedure volume. Multivariable nested regression was used to determine variables that predict perfect inpatient care, which would be assessed based on death, sepsis, postoperative infection, thromboembolic events, venous thrombosis, hematoma, blood transfusion, and length of stay below average | Hospital arthroplasty volume ranged from 0 to 11,758 procedures. Overall, hospital PICI scores increased as arthroplasty volume increased. In multivariable nested regression analysis, procedure volume (odds ratio [OR] for the highest quartile compared with the lowest quartile, 2.116 [95% confidence interval (CI), 1.883 to 2.378]) and lower patient acuity (OR, 2.450 [95% CI, 2.429 to 2.472]) were independently associated with better PICI scores. Value increased as hospital procedure volume increased. |
| Inneh IA, Clair AJ, Slover JD, et al | Retrospective cohort study (3) | The purpose of this paper is to analyze the factors that influence discharge destination following TJA. | There were a total 7924 TJA cases, of which 4836 (61%) were female, 785 (10%) were of low socioeconomic status, and 2770 (35%) were of nonwhite race/ethnicity. | Data on TJA patients at a single institution between 2011 and 2014 was analyzed. Evaluation of socioeconomic, geographic, and racial/ethnic factors associated with discharge destination to either home or institution was performed. | A total of 5088 (64%) cases were discharged to home and 2836 (36%) cases were discharged to institution. Significant predictors of discharge to an institution include low and middle SES (odds ratio [OR]: 1.27, 95% confidence interval [CI]: 1.02-1.57, p= .029; and OR: 1.26, 95% CI: 1.10-1.44, p= .001), age (OR: 1.05, 95% CI: 1.049-1.060, p< .001), female gender (OR: 1.69, 95% CI: 1.52-1.89, p< .001) and TKA procedure (OR: 1.48, 95% CI: 1.33-1.64, p< .001). Patients of nonblack race/ ethnicity were more likely to be discharged home (white OR: 0.84, 95% CI: 0.72-0.98, p=.027; other OR: 0.80, 95% CI: 0.67-0.95, p=.009). |
| Koltsov JCB, Marx RG, Bachner E, et al | Retrospective cohort study (3) | Use quantitative methods to derive meaningful, risk-based categories for hospital and surgeon total hip arthroplasty (THA) volume based on relationships with mortality, complications, and revision. | Information regarding all primary, unilateral THA procedures (ICD-9-CM [International Classification of Diseases, Ninth Revision, Clinical Modification] procedure code 81.51) performed in residents of New York State between 1997 and 2014 was obtained from the Statewide Planning and Research Cooperative System (SPARCS) inpatient databases (n = 187,557) | Using New York statewide patient data (1997 to 2014; n = 187,557), authors derived risk-based hospital and surgeon-volume categories for primary THA based on relationships with 90-day complications and mortality and 2-year revision. | More than 35% of THA cases in New York State were conducted by surgeons performing ≤1 THA/month (0 to 12 THA/year), and these were associated with a 2 to 2.5-fold increase in the risk for complications, mortality, and revision relative to higher-volume surgeons. Similarly, 15% of THA cases in New York State were conducted in hospitals performing ≤1 THA/week (0 to 11 or 12 to 54 THA/year), and these were associated with a nearly 1.5-fold increase in complications and between a 4 and 6-fold increase in mortality. |
| Lan RH, Kamath AF | Retrospective cohort study (3) | The purpose of this investigation was to better understand the socioeconomic factors that influence hospitalization and post-discharge metrics after joint replacement to identify key areas of improvement in delivering orthopaedic care. | A total of 2869 consecutive patients who underwent total hip (THA) or knee arthroplasty (TKA) were included within the study. 1832 (63.9%) of patients were female, 1349 (47%) were white, 1391 (48.5%) were black, 49 (1.7%) were Asian and 80 (2.9%) were classified as other. | The authors analyzed an institutional administrative data set from an academic arthroplasty referral center to identify patients who underwent THA or TKA from 2007 to 2015. Univariate and stepwise forward logistic regression analyses were used to determine the relationship between the independent variables of gender, race, and insurance and the dependent variables of institutional care and prolonged LOS. | Females (odds ratio [OR] = 2.07, 95% confidence interval [CI] 1.74-2.46), minorities (OR = 2.11, 95% CI 1.78-2.51), and non-private insurance holders (OR = 1.56, 95% CI, 1.26-1.94) were more likely to be assigned to institutional care after discharge. Minorities (OR = 1.45, 95% CI 1.24-1.70) and non-private insurance holders (OR = 1.43, 95% CI 1.16-1.77) are more likely to exhibit longer length of stay. Mean charges were higher for males when compared to females ($80,010 vs $74,855, P < 0.001), as well as total costs ($19,910 vs $18,613; P < 0.001). Whites are more likely to hold private insurance than blacks (21.1% vs 11.3%, P < 0.001), as well as Medicare (32.5% vs 17.8%; P < 0.001) and managed care (26.8% vs 13.6%; P < 0.001). Blacks are more likely to hold Medicaid than whites (28.3% vs 10.5%) as well as managed Medicare (27.1% vs 6.6%; P < .001). |
| Laucis NC, Chowdhury M, Dasgupta A, et al | Retrospective cohort study (3) | What qualifies as a "high-volume" hospital and what is the relationship between hospital volume and complication rate after THA and TKA? | Patients who underwent elective primary THA and TKA were grouped by hospital | Data from the National (Nationwide) Inpatient Sample (2000 to 2012) were used to quantify trends in total hip arthroplasty (THA) and total knee arthroplasty (TKA) volume. County geographic and population data were obtained from the U.S. Census, and the distances between hospitals and the centroids of counties were calculated. Risk-standardized surgical complication rates for hospitals (2009 to 2012) were obtained from Medicare Hospital Compare and grouped by hospital volume | In 2012, 65.5% of the arthroplasties were performed in high-volume hospitals (≥400 arthroplasties annually), and 26.6% of the arthroplasties were performed in very high-volume hospitals (≥1,000 procedures annually). The proportion of arthroplasties performed in low-volume hospitals (<100 arthroplasties annually) shrank from 17.9% to 5.4%. Very high-volume hospitals had the lowest complication rates (2.745 per 100; 95% confidence interval [CI], 2.56 to 2.93), and low-volume hospitals had the highest complication rates (3.610 per 100; 95% CI, 3.58 to 3.64; p < 0.0001) (odds ratio, 1.327; 95% CI, 1.26 to 1.40). |
| Lavernia CJ, Lee D, Sierra RJ, et al | Retrospective cohort study (3) | To examine the association between race/ethnicity and insurance type and the preoperative status of patients undergoing joint arthroplasty surgery | A total of 739 primary total hip or knee arthroplasties performed on 573 patients (293 hip and 280 knee). The mean age of the cohort was 62.7 years (standard deviation [SD] = 14.1). Of the total number of patients, 361 (63.2%) were females, and 328 (57.3%) were of Hispanic origin. Within the Hispanic subgroup, 215 were Cuban/Cuban American, 82 were South or Central American, 13 were Puerto Rican, 5 were Mexican/Mexican American, and 13 were Spanish. In the present analysis, participants were classified as: Hispanic whites, 300 patients (52.4%); non-Hispanic whites, 157 (27.4%); Hispanic blacks 28 (4.9%); and non-Hispanic blacks, 88 (15.4%). Patients were also classified by type of insurance coverage, which was approximately evenly distributed across Medicare (38%), Medicaid/indigent (31%), and private insurance (31%) | From August 1992 to January 2000, a consecutive series of patients with a diagnosis of end-stage arthritis who underwent primary THA or TKA were retrospectively reviewed. Statistical analysis was performed on PROMs after surgery. A 2-way analysis of variance (ANOVA) with interaction was used to assess the joint influence of race/ethnicity and insurance. | Non-Hispanic whites had lower preoperative pain and WOMAC scores and higher Quality Well Being Index and SF-36 scores compared with other racial/ethnic subgroups. Patients with Medicare/private insurance had better preoperative scores relative to patients with Medicaid or no insurance. Racial/ethnic status was generally more strongly associated with preoperative status than was insurance type. Hispanics, blacks, and patients without Medicare or private health insurance reach arthroplasty surgery with lower preoperative functional and health status |
| Li Y, Ying M, Cai X, et al | Prospective cohort study (2) | What are the longitudinal trends in the use of post-acute care (PAC) after hip and knee replacement surgery, and in gaps among Medicare patients of different socioeconomic status, under current Medicare payment reforms? | This sample consisted of all discharges of Medicare fee for service beneficiaries who received total or partial hip or total knee replacement, who were 65 years or older, between 01/01/2013 -12/31/2016. The sample included 1,302,256 patients, 64.3% of which were woman. The average age was 75.4 years old. Of the cohort, 90.8% were Medicare-only benefit shear ease, 4.6% were dual-eligible patients with full benefits, and 4.5% were dual-eligible patients with partial benefits. | The authors analyzed the National Master Beneficiary Summary Files, Medicare Provider Analysis Review Claims, several PAC assessments files, Inpatient Rehab Facility Patient Assessment Instrument files, and health Outcome and Assessment Information Sets for data between 1/1/13-12/31/16. Outcomes included whether the patient was discharged to institutional post-acute care (i.e. SNF [skilled nursing facility], IRF [inpatient rehabilitation facility], LTCH [long-term care hospital]), rehospitalization within 30 days of discharge, total payments for all 30-day readmissions, and 90-day readmissions with corresponding total payments. Medicare and Medicaid dual eligible status of beneficiaries was also tracked. | Compared with Medicare-only patients, dual-eligible patients were slightly older, less likely to be non-Hispanic white, more likely to have congestive heart failure, more likely to have uncomplicated or complicated diabetes, more likely to have renal failure, more likely to be discharged from hospitals with lower volumes for replacement surgery. From 2013 to 2016, institutional PAC discharge decreased for all groups. For Medicare-only patients, the rate went from 43.7% to 32.5%. For dual-eligible patients, the rate went from 70.1% to 61.5% for partial-benefits patients and 62.3% for full benefits patients.  Home discharges increased for all groups: from 56.4% to 67.5% in Medicare-only patients and from 29.8% to 38.1% for both dual-eligible groups.  Unadjusted 30-day readmission rates decreased from 10.6% to 7.8% for Medicare-only patients, from 14.6% to 12.0% for dual-eligible patients with full benefits, and from 18.4% to 15.6% for dual-eligible patients with partial benefits. Compared with Medicare-only patients and within 90 days of hospital discharge, dual-eligible patients with partial benefits were 24% (in 2013) to 37% (in 2016) more likely to be readmitted (p=.006 for 2016, p<.001 for both). From 2013-2016, among patients discharged to SNFs, 65.7% to 72.3% of Medicare-only patients were discharged to 4 or 5-star SNFs, while 56.2% to 62.6% of dual-eligible patients with full benefits and 52.5% to 60.9% of dual-eligible patients with partial benefits were discharged to 4-star or 5-star SNFs. From 2013 to 2016, unadjusted proportion of patients successfully discharged to community after a SNF stay was flat at 80.5% for Medicare-only patients, 59.8% for dual-eligible patients with full benefits, and 50.0% for dual-eligible patients with partial benefits. Compared with Medicare-only patients in a SNF, dual-eligible patients with full or partial benefits were half as likely to be successfully discharged to community (p<.001), which persisted over time. Compared with Medicare-only patients, dual-eligible patients with full benefits had a longer SNF LOS by approximately 12 to 15 days (p<.001 for both cases, p<.001 for trend); dual-eligible patients with partial benefits had a longer SNF LOS by approximately 5 to 8 days (p<.001 for both cases, p<.001 for trend). |
| Maman SR, Andreae MH, Gaber-Baylis LK, et al | Retrospective cohort study (3) | The aim of this study is to determine whether having the insurance type Medicaid can predict in-hospital morbidity and mortality. | The total number of patients included was 922,819. 29,692 of these (3.2%) had Medicaid insurance. 63.2% of all patients were female. 8.1% were black, 7.9% were Hispanic, and 76.8% were white. | The primary outcome measure was in-hospital mortality. Regression models were used to test the hypothesis in patients in the State Inpatient Database (SID, 2007-2014) from five states who underwent primary TKA. States with databases analyzed were Florida, New York, Marylan, Kentucky, and California. Secondary outcomes were hospital length of stay (LOS) and postoperative complications, including intraoperative/procedure related, infectious, wound, pulmonary, urinary, gastrointestinal, cardiovascular and systemic | Medicaid patients had greater odds of in-hospital mortality (odds ratio [OR]: 1.73; 95% CI: 1.01–2.95; p≤.05), greater odds of any postoperative complications (OR: 1.25; 95% CI: 1.18–1.33; p≤.005), experience longer lengths of stay (OR: 1.09; 95% CI: 1.08–1.10; p≤.005) and higher total charges (OR: 1.03; 95% CI: 1.02–1.04; p≤.005). |
| Manley M, Ong K, Lau E, et al | Retrospective cohort study (3) | What is the association between hospital and surgeon volume and TKA survivorship? | Elderly population that underwent TKA was assessed using 1997 to 2004 Medicare data | Kaplan-Meier method and Cox regression were used to determine implant survivorship and hazard ratios associated with procedure volume at 0.5, 2, 5, and 8 years | The TKA patients in lowest-volume hospitals (1-25 procedures) had a higher risk of revision at 5 and 8 years compared with those operated on in highest-volume hospitals (>200 procedures) (adjusted odds ratio: 1.57 and 1.52, respectively). Surgeon volume was not significantly correlated with implant survivorship. Our findings suggest that TKA patients at low-volume hospitals have a greater revision risk at medium-term follow-up, but not in the short term |
| Martin CT, Callaghan JJ, Liu SS, et al | Retrospective cohort study (3) | Is there a disparity in preoperative patient factors between insurance typed in total joint arthroplasty? | 1312 consecutive patients who underwent elective total hip or total knee arthroplasty were included, of which 36% had private insurance, 47% had Medicare, 5% had Medicaid, and 13% had Iowa Care. | Patients who underwent elective total hip or total knee arthroplasty and also had available Short Form 36 (SF-36) and Western Ontario and McMaster University Osteoarthritis Index surveys were included and stratified into groups based on insurance type (Iowa Care [state-run insurance program for patients who are indigent], Medicaid, Medicare, or private insurance). Demographics and functional data were collected for analysis. Analysis was performed to identify independent predictors of preoperative functional status based on the above surveys. | Compared with Medicare or private insurance patients, those with Iowa Care or Medicaid has significantly lower SF-36 and Western Ontario and McMaster University Osteoarthritis Index scores in all categories (p<.05 for each comparison). Iowa Care and Medicaid patients had a higher incidence of current smoking, higher mean body mass index, and travelled an average of 29 to 30 miles farther for access to care (p<.05 for each). Payer type was an independent predictor of preoperative Short Form 36 and Western Ontario and McMaster University Osteoarthritis Index functional scores in the analysis (p<.02). |
| Menendez ME, Ring D, Barnes CL | Retrospective cohort study (3) | This study aimed to investigate whether inpatient dislocation after THA could be associated with patient and hospital characteristics. | All adult patients undergoing elective primary THA were considered, and the population was further narrowed by identifying those who sustained a hip dislocation during index hospitalization. The final number was 2,173. | Discharge records from the Nationwide Inpatient Sample (2002-2011) were used for the data source. Temporal trends were assessed, and multivariable logistic regression modeling was used to identify factors associated with dislocation. | The in-hospital dislocation rate increased 2002 to 2011, despite a downward trend in length of stay (p< .001). Patient characteristics associated with the occurrence of dislocation were black (p<.001) or Hispanic (p=.001) race/ethnicity, lower household income (p< .001), and Medicaid insurance (p=.034). Comorbidities associated with dislocation included hemiparesis/hemiplegia (p< .001), drug use disorder (p=.02), chronic renal failure (p< .001), psychosis (p=.027), and obesity (p< .001). Age, sex, and alcohol use disorder did not affect the dislocation risk. Dislocations were less likely to occur at teaching hospitals (p< .001) and in the South (p=.002). |
| Oronce CI, Shao H, Shi L | Retrospective cohort study (3) | The purpose of this investigation was to identify disparities in elective total hip arthroplasty (THA) readmissions based on race, socioeconomic status and type of insurance. | A total of 58,777 patients who were discharged from a California Hospital after undergoing elective THA were included. 85.24% were white, 4.83% were black, 7.47% were Hispanic and 2.55% were Asian. The median age for black and Hispanic patients was 62 and 63 compared to 67 years old for white and Asian patients. 41% of African Americans, 26% of Hispanics and 14% of whites were within the lowest quartile for socioeconomic status (P < 0.001). 10% of blacks and 9% of Hispanics were enrolled in Medicaid (P < 0.001) and 5% were uninsured (P < 0.001) | The authors analyzed data from The Healthcare Cost and Utilization Project's State Inpatient Database from California to identify index hospitalization for elective primary THA and rehospitalization within 30 days of discharge. Multivariate logistic regression was used to examine differences in all-cause readmission within 30 days by race, socioeconomic status and insurance. | The overall rate of unplanned 30-day all-cause readmissions after elective primary THA was 4.6%. African American (Odds Ratio [OR] = 1.38, 95% Confidence Interval [CI] 1.16–1.64) and Hispanic (OR = 1.16, 95% CI 1.00–1.34) patients had a higher risk of readmission than white patients after THA when accounting for comorbidities and hospital factors. Lower socioeconomic status was associated with higher odds of readmission within 30 days (OR = 1.24, 95% CI 1.10–1.39). Compared with private insurance, Medicare (OR = 1.26 95% CI 1.13–1.43), Medicaid (OR = 1.86 95% CI 1.49–2.32), and uninsured status (OR = 1.31, 95% CI 1.01–1.69) were also associated with increased readmission risk. |
| Plate JF, Ryan SP, Goltz DE, et al | Retrospective cohort study (3) | Determine whether Medicaid patients have increased resource utilization (including 90-day emergency department [ED] visits and readmissions) compared to Medicare or commercial insurance carriers. | A total of 3674 primary THA patients were included in the analysis (including 116 with Medicaid, 1713 with Medicare, and 1845 with other insurance providers). Medicaid patients had significantly higher ASA scores (P < .001) and BMI (P < .001), with corresponding increase in procedure duration (115 vs 99 vs 105 minutes; P < .001) | The institutional database was retrospectively queried for primary THAs from 2013 to 2017 based on Current Procedural Terminology codes and patients undergoing revision surgery were excluded. Demographic information including age, sex, and body mass index (BMI) and medical comorbidities including American Society of Anesthesiologists (ASA) scores were evaluated. Patients were stratified by insurance type and length of stay (LOS), and 90-day ED visits and 90-day readmissions were assessed in univariable and multivariable analysis | Medicaid patients had a prolonged LOS (2.5 vs 2.5 vs 1.5 days; P < .001) compared with other insurances, but similar to Medicare patients. Following discharge, in multivariable analysis controlling for age, BMI, and ASA score, Medicare patients were significantly more likely to return to the ED (odds ratio, 3.15; 95% confidence interval, 1.88-5.27; P < .001) and be readmitted (odds ratio, 2.46; 95% confidence interval, 1.26-4.81; P = .009). Medicaid patients represent a higher risk cohort with increased resource utilization perioperatively, including longer LOS, and more 90-day ED visits and readmissions. |
| Singh JA, Cleveland JD | Retrospective cohort study (3) | Do health insurance type and income affect outcomes after THA? | In a National Inpatient Sample (NIS) cohort of 4,116,485 primary THA patients, mean age was 65.5 years, 57% were female, 87% were white, and 83% underwent surgery for osteoarthritis | Cox-regression analysis was performed on National Inpatient Sample (NIS) patients from 1998-2014. Analysis was performed to determine if there is any association between Medicaid/Medicare insurance type and income with post-THA health care utilization, outcomes, and complications. | Compared to private insurance, patients with Medicaid had significantly higher hazard ratio (HR) (95% confidence interval (CI)) for hospital charges above the median, 1.18 (1.15, 1.21); discharge to a rehabilitation/inpatient facility, 1.67 (1.62, 1.72); length of hospital stay > 3 days, 1.62 (1.58, 1.67); and in-hospital post-operative complications including infection, 1.70 (1.47, 1.97); transfusion, 1.13 (1.09, 1.16); revision, 1.55 (1.32, 1.82); and mortality, 1.89 (1.35, 2.63). Results were similar for those with Medicare payer status. Compared to the highest quartile, the lowest income quartile was associated with significantly higher HR (95% CI) of hospital charges above median, 1.43 (1.41, 1.45), and a lower HR of discharge to a rehabilitation/inpatient facility, 0.78 (0.77, 0.79); hospital stay > 3 days, 0.82 (0.80, 0.83); infection, 0.57 (0.50, 0.65); and transfusion, 0.80 (0.79, 0.82). |
| Singh JA, Kwoh CK, Boudreau RM, et al | Retrospective cohort study (3) | To examine the relationship between hospital procedure volume and surgical outcomes following elective primary total hip arthroplasty/total knee arthroplasty (THA/TKA) | All patients who underwent elective primary THA/TKA in Pennsylvania were included. Mean age was 69 years. 42.8% and 35%, respectively, were men in THA and TKA cohorts. | Using the Pennsylvania Health Care Cost Containment Council database, all patients who underwent elective primary THA/TKA in Pennsylvania were identified. Hospitals were categorized according to the annual volume of THA/TKA procedures, as follows: ≤25, 26-100, 101-200, and >200. The 30-day complication rate and 30-day and 1-year mortality rates were assessed by logistic regression models, adjusted for age, sex, race, insurance type, hospital region, 3M All Patient Refined Diagnosis Related Group risk of mortality score, hospital teaching status, and bed count. | Compared with patients whose surgeries were performed at very-high-volume hospitals (>200 procedures/year), patients who underwent elective primary THA procedures at hospitals with a very low volume (≤25 procedures/year), a low volume (26-100 procedures/year), or a high volume (101-200 procedures/year) had higher multivariable-adjusted odds ratios (ORs) for venous thromboembolism (OR 2.0, 95% confidence interval [95% CI] 0.2-16.0), OR 3.4 [95% CI 1.4-8.0], and OR 1.1 [95% CI 0.3-3.7], respectively) and 1-year mortality (OR 2.1 [95% CI 1.2-3.6], OR 2.0 [95% CI 1.4-2.9], and OR 1.0 [95% CI 0.7-1.5], respectively). Among patients ages ≥65 years who underwent elective primary TKA at very-low-volume, low-volume, and high-volume hospitals, the ORs for 1-year mortality were significantly higher (OR 0.6 [95% CI 0.2-2.1], OR 1.6 [95% CI 1.0-2.4], and OR 0.9 [95% CI 0.6-1.3], respectively), compared with very-high-volume hospitals. |
| Singh JA, Lewallen DG | Prospective observational study (2) | To assess whether income is associated with patient-reported outcome measures (PROMs) after primary TKA | 7, 139 primary TKAs from the Mayo Clinic Total Joint Registry was assessed. | There were 7139 primary TKAs at 2 years postop and 4,234 TKAs available for follow up at 5 years postop. This prospectively collected data was analyzed using multivariate-adjusted logistic regression. | At 2-year follow-up, compared to income > US$45, 000, lower incomes of ≤ US$35, 000 and > US$35, 000 to 45, 000 were associated (1) significantly with moderate to severe pain with an odds ratio (OR) 0.61 (95% CI 0.40 to 0.94) (P = 0.02) and 0.68 (95% CI 0.49 to 0.94) (P = 0.02); and (2) trended towards significance for moderate to severe activity limitation with OR 0.78 (95% CI 0.60 to 1.02) (P = 0.07) and no significant association with OR 0.96 (95% CI 0.78 to 1.20) (P = 0.75), respectively. At 5 years, odds were not statistically significantly different by income, although numerically they favored lower income. In multivariable-adjusted analyses, overall improvement in knee function was rated as 'better' slightly more often at 2 years by patients with income in the ≤ US$35, 000 range compared to patients with income > US$45, 000, with an OR 1.9 (95% CI 1.0 to 3.6) (P = 0.06) |
| Skinner J, Zhou W, Weinstein J | Retrospective cohort study (3) | The purpose of this study is to investigate the associations between income, total knee arthroplasty (TKA), and underlying rates of knee osteoarthritis. | A total of 27.5 million patients were analyzed from the US Medicare claims database, and a total of 1926 were analyzed from the NHANES III. | The 2000 US Medicare claims database was used to measure incidence of total knee arthroplasty by race, ethnicity, postal code income, and region. The National Health and Nutrition Examination Survey (NHANES III) for persons sixty years or older with radiographic and clinical evidence of osteoarthritis was also used. | Age-adjusted rates of TKA in the high-income quintile were no higher than those in the low-income group. Access to care was better for high-income groups (18.5% higher than the lowest quintile). Racial disparities in arthroplasty were significant, with black patients receiving less TKAs than their white counterparts (p<.001). There was no evidence of an income gradient for clinical and radiographic measurements of arthritis, other than a significant negative association between income and pain on passive motion (p<.05). |
| SooHoo NF, Farng E, Zingmond DS | Retrospective cohort study (3) | The purpose of this investigation was to identify the characteristics of patients who undergo total hip replacement (THR) at high-volume hospitals and their differences from those who receive care at low-volume hospitals. | A total of 138,399 patients who underwent THR surgery in one of 399 California hospitals between 1995 and 2005. There were 53,215 procedures (38%) performed at hospitals with low (6221) or intermediate (46,994) surgical volumes. 85,184 (62%) procedures occurred at higher-volume hospitals. The average age of all patients was 66.16 ± 12.92. 79,514 (57%) of patients were female. 117,107 (85%) were white, 6,051 (4%) were black, 9,368 (7%) were Hispanic and 3006 (2%) were Asian. | The authors analyzed the California Office of Statewide Health Planning and Development (OSHPD) database to identify patients who underwent THR surgery between 1995 and 2005. Inclusion criteria included patients who underwent primary THR in a California hospital. Exclusion criteria included patients with infection, pathologic fracture or undergoing revision arthroplasty. Logistic regression models were created to examine the association among patient characteristics including race/ethnicity, income, age, and the Charlson comorbidity index of patients and the likelihood of undergoing surgery at low-, intermediate-, and high-volume hospitals. | There were 160 (40%) low-volume and 160 (40%) intermediate-volume hospitals included within the study. The remaining 20% of the hospitals made up the group of 79 high-volume centers. The average surgical volume was 3.5 cases per year per hospital at low-volume hospitals. The average was 26.7 cases per year per hospital at intermediate-volume centers, and 98.0 cases per year per high-volume hospital. High volume hospitals had a higher percentage of Caucasian patients (87%) than intermediate (83%) or low-volume hospitals (68%). Conversely, the proportion of patients in the lowest quartile of income was greater at low volume (9%) and intermediate-volume (5%) hospitals than at high-volume centers (3%). Hispanic patients had the highest Relative Risk Ratio (RRR) for being treated at either a low-volume (RRR = 3.52, 95% Confidence Interval [CI] 2.61-4.74, P < 0.001) or intermediate volume hospital (RRR = 1.60, 95% CI 1.24-2.06, P < 0.001) when compared to Caucasian patients. Black (RRR = 1.78, 95% CI, 1.08-2.92; p = 0.023) and Asian (RRR = 1.77, 95% CI, 1.00-3.22, P = 0.048) patients also had a high risk of being treated in a low volume hospital compared to Caucasian patients. Patients within the lowest income group were at increased risk of being treated at either a low-volume (RRR = 3.19, 95% CI 1.89-5.37, P < 0.001) or intermediate volume (RRR = 1.80, 95% CI 1.09-2.98, P = 0.02) hospital compared to patients within the highest income group. |
| SooHoo NF, Zingmond DS, Ko CY | Retrospective cohort study (3) | The purpose of this study is to identify the characteristics of patients who undergo total knee arthroplasty (TKA) at high-volume hospitals and their differences from those who receive care at low-volume hospitals. | 222,684 patients' data was analyzed from the study period. Primary TKA was performed at 413 hospitals. There were 165 low-volume and 165 intermediate-volume hospitals, comprising of 40% of the total. | Patients who underwent TKA in California from 1991-2001 had their discharge data analyzed. Hospitals were classified into tiers of surgical volume (low [bottom 40th percentile], intermediate [middle 40th percentile], high [top 20th percentile]). Separate logistics regression models were created to examine the relationships between race/ethnicity, insurance status, and the utilization of high-volume and low-volume hospitals. Logistic regression models corrected for covariates including age, gender, and comorbidity. | Patients who were not belonging to the Caucasian race/ethnicity had a higher relative risk ratio for being treated at a low-volume center, including Black (p=.02), Hispanic (p<.001), and Asian/Pacific Islander (p<.001) ethnic groups. Medicaid insurance was an independent predictor of treatment at low-volume hospitals. |
| Starring H, Waddell WH, Steward W, et al | Retrospective cohort study (3) | To compare PROs after TKA in different insurance groups after adjusting for proposed risks. | Medicare and Medicare Advantage patients were older (p < 0.001) and had more comorbidities (p = 0.001) than commercial patients. Otherwise, there were no statistically significant differences between Medicare Advantage patients and either commercially insured or Medicare patients. | Demographics and clinical data were abstracted from medical records of 302 patients who underwent TKA performed by a single surgeon at a university-based orthopaedic practice during 2013 to 2017. Differences in PROs between commercially insured, Medicare Advantage, and Medicare patients during the 6 months following surgery were evaluated while controlling for demographics, clinical data, and baseline PRO scores. | During the first 3 months following TKA, patients in all three groups experienced similar rates of recovery. At 6 months after surgery, outcomes began to diverge by insurance group. Medicare patients reported significantly less ability to perform activities of daily living (78.6 vs. 63.2; p = 0.001), worse physical function (39.6 vs. 44.9; p = 0.003), and more pain interference (57.9 vs. 52.4; p = 0.018) at day 180 than commercially insured patients. |
| Veltre DR, Sing DC, Yi PH, et al | Retrospective cohort study (3) | Evaluate the effect of patient insurance status on complications after primary elective THA using the Nationwide Inpatient Sample | About 515,037 patients (53.7% Medicare, 40.1% private insurance, 3.9% Medicaid/uninsured, and 2.2% other) were included, who underwent elective THA. Privately insured patients had fewer medical complications (odds ratio, 0.80; P < 0.001), whereas patients with Medicaid or no insurance demonstrated no notable difference (odds ratio, 1.03; P = 0.367) compared with Medicare patients | All patients undergoing primary elective THA from 1998 to 2011 were included. Patient demographics, comorbidities, and complications were collected and compared based on insurance type. Multivariable logistic regression and a matched cohort analysis were performed. | Similar trends were found for both surgical complications and mortality, favoring lower complication rates for privately insured patients. Furthermore, patients with private insurance tend to go to higher volume hospitals for total hip replacement surgery compared to those with Medicare insurance. Patients with government-sponsored insurance (Medicare or Medicaid) or no insurance have higher risk of medical complications, surgical complications, and mortality after primary elective THA compared with privately insured patients. Insurance status should be considered an independent risk factor for stratifying patients before THA procedures |
| Veltre DR, Yi PH, Sing DC, et al | Retrospective cohort study (3) | The purpose of this study was to evaluate the effect of patient insurance status on the in-hospital complication rates following total knee arthroplasty. | A total of 1,352,505 patients (Medicare, 57.8%; private insurance, 35.6%; Medicaid/uninsured, 3.1%; other, 3.3%; unknown, 0.2%) fulfilled the inclusion criteria. | Data was obtained from the Nationwide Inpatient Sample (NIS, 2004-2011). Patient demographics and comorbidities were analyzed and stratified by insurance type between Medicare and private insurance. Primary outcome was in-hospital complication rate. | Patients with Medicare showed significantly higher risk of mortality (relative risk [RR], 1.34; p<.001), wound dehiscence (RR, 1.32; p<.001), central nervous system complications (RR, 1.16; p=.030), and gastrointestinal complications (RR, 1.13; p<.001). Private insurance patients showed a higher risk of cardiac complications (RR, 0.93; p=.003). |
| Weiner JA, Adhia AH, Feinglass JM, et al | Retrospective cohort study (3) | The purpose of this investigation was to evaluate which patient characteristics are associated with extended length of stay (eLOS) of greater than 2 days and nonhome discharge in patients undergoing total hip arthroplasty (THA). | A total of 41,832 patient who underwent primary THA between 2016 and 2018 were included within the study. 81.4% of patients were non-Hispanic white and 92.6% had osteoarthritis (OA) as a principal diagnosis. 55.6% were female, 60% had a Charlson comorbidity index (CCI) of 0, and 49.1% of THAs were completed at an academic institution. Medicaid or uninsured patients represented 5.1% of the included population and 3.9% of patients resided in a low-income zip code. | The authors analyzed the Illinois Hospital Association COMPdata administrative database from 151 Illinois nonfederal hospitals to evaluate patients who underwent primary THA from 2016 to 2018. LOS was classified as 2 days or less, eLOS if greater than 2 days, and very extended LOS (veLOS) if greater than 4 days. Chi square tests were used to determine the significance of associations between home discharge and LOS (grouped as LOS 3 or 4 days and veLOS) and patient sex, age, race and ethnicity, Illinois region, low-income zip code, Medicaid/uninsured, time period, obesity, CCI, principal diagnosis, facility BPCI status, facility academic status, facility volume quartile, and Illinois region. A multiple Poisson regression model was estimated to test the simultaneous association between patient and hospital characteristics and nonhome discharge, eLOS, and veLOS. | 74.7% were discharged home with 64.0% having a LOS of 2 days or less. Only 36.0% of patients had eLOS and 5.3% had veLOS. Very few of those (7.5%) whose LOS was 2 days or less were not discharged home. That was the opposite trend seen for those staying 3 to 4 days (53.7%) and 5 or more days (77.0%) after THA, as they were more likely to be discharged to a nonhome facility. Females represented 65.8% of those with a LOS of 3 or 4 days. Females were 35% more likely than males (Incidence Rate Ratio [IRR] = 1.35, 95% CI 1.29-1.40) to experience eLOS. Those aged 75 years and older were 47% more likely to have eLOS than those aged 65 to 74 years (IRR = 1.47, 95% CI 1.40-1.53). Patients with a CCI of 1 were 25% more likely to have eLOS (IRR = 1.25, 95% CI 1.20-1.30). Patients with a CCI of 3 or greater were 59% more likely to have eLOS (IRR = 1.59, 95% CI 1.48-1.70). 41.5% of patients with veLOS were older than 75, compared to 21.9% in the overall cohort. Those older than 75 years were 59% more likely (IRR = 1.59, 95% CI 1.42-1.78) than those aged 65 to 74 years to have veLOS. Non-Hispanic blacks were 42% more likely than non-Hispanic whites to have veLOS (IRR = 1.42, 95% CI 1.19-1.70). Medicaid and uninsured patients were 67% more likely to have veLOS than those with other types of insurance (IRR = 1.67, 95% CI 1.33-2.09). Those with CCI of 3 or more were more than 226% more likely to have veLOS than those with CCI of 0 (IRR = 3.26, 95% CI 2.87-3.71). Those aged 75 years, or more were 88% more likely than those 65 to 74 years old to have nonhome discharge (IRR = 1.88, 95% CI 1.77-2.00). Those with a CCI score of 3 were 77% more likely to experience nonhome discharge when compared to those with a CCI of zero (IRR = 1.77, 95% CI 1.63-1.91). Medicaid or uninsured patients were 30% more likely to have a nonhome discharge (IRR = 1.30, 95% CI 1.13-1.49), and non-Hispanic black ethnicity, which was 11% more likely to be associated with nonhome discharge than non-Hispanic white ethnicity (IRR = 1.11, 95% CI 1.00-1.23). |
| White RS, Sastow DL, Gaber-Baylis LK, et al | Retrospective cohort study (3) | The purpose of this study was to examine differences in readmission rates by insurance payer, race, ethnicity and income status. | A total of 274,851 adult patients who had undergone THR surgery in California, Florida or New York were included. 154,695 (56.3%) of patients had Medicare, 9,099 (3.3%) had Medicaid and 101,897 (37.1%) had private insurance. 227,354 (82.7%) of all patients were white, 15,644 (5.8%) were black and 14,718 (5.7%) were Hispanic. 155,597 (56.6%) of all patients were female. | The authors analyzed the California, Florida and New York State Inpatient Databases (SID), Healthcare Cost and Utilization Project, Agency for Healthcare Research and Quality to identify patients who underwent THR from 2007 to 2011. Inclusion criteria included patients who had undergone THR surgery and were old than 18 years of age. Exclusion criteria included those with missing data on gender, experienced inpatient mortality during their index hospital stay, had missing information on hospital length of stay (LOS) or days to readmission data, had insufficient follow-up time or lacked primary insurance status information. Categorical variables were compared using Chi squared tests or Fischer's exact tests and continuous variables were compared using analysis of variance (ANOVA) or Kruskal-Wallis tests. Logistic regression analysis was completed to evaluate the effect of racial and socioeconomic disparities on 30 and 90-day readmissions. | Within 30 days, 15,273 (5.6%) patients were readmitted, including 7.7% of Medicaid patients, 6.8% of Medicare patients, and 3.5% of patients with private insurance. After 30-day readmission, 271 patients (1.8%) died during their hospitalization. When compared to patients who were not readmitted within 30 days, patients readmitted within 30 days were more likely to be older (69.62 ± 13.18 vs. 66.03 ± 12.30, P < 0.0001), be Black (7.4% vs. 5.6%, P < 0.0001) or Hispanic (6.0% vs. 5.3%, P < 0.0001), live in the poorest quartile of median household income in their respective ZIP code (20.3% vs. 16.7%, P < 0.0001), have Medicare (69.1% vs. 55.5%, P < 0.0001) and Medicaid (4.6% vs. 3.2%, P < 0.0001) as insurance providers. Patients readmitted within 30-days had an initial hospital course with a longer length of stay (4 days vs. 3 days, P < 0.0001) and have greater total hospital charges ($69,471 vs. $63,474, P < 0.0001) than those not readmitted up to 30 days postoperatively. Additionally, readmitted patients were more likely to have suffered cardiovascular, pulmonary, infectious, and intraoperative complications during their initial postoperative hospitalization (P < 0.0001 for each). The most common reasons for 30-day readmissions were wound infection (15.7% Private insurance vs. 19.3% Uninsured), atrial fibrillation (5.9% Private insurance vs. 18.4% Medicare), urinary tract infection (6.3% Private insurance vs. 13.3% Medicare), and pneumonia (3.4% Private insurance vs. 6.9% Medicare). Within 90 days, 28,075 (10.2%) patients were readmitted, including 14.5% of Medicaid patients, 11.9% of Medicare patients, and 7.3% patients with private insurance. After 90-day readmission, 432 (1.5%) died during their hospitalization. When compared to patients who were not readmitted within 90 days, patients readmitted within 90 days were more likely to be older (68.39 ± 13.45 vs. 65.98 ± 12.22, P < 0.0001), be Black (7.0% vs. 5.5%, P < 0.0001) or Hispanic (5.7% vs. 5.3%, P < 0.0001), live in the poorest quartile of median household income in their respective ZIP code (19.7% vs. 16.6%, P < 0.0001), have Medicare (65.7% vs. 55.2%, P < 0.0001) and Medicaid (4.7% vs. 3.2%, P < 0.0001) as insurance providers. Patients readmitted within 90-days had greater total hospital charges ($68,767 vs. $63,236, P < 0.0001) than those not readmitted up to 90 days postoperatively. The most common reasons for 90-day readmission compared to private insurance were atrial fibrillation (5.5% Private Insurance vs. 16.9% Medicare), urinary tract infection (4.5% Private insurance vs. 12.0% Medicare), wound infection (9.3% Private insurance vs. 10.7% Other Insurance), and pneumonia (2.7% Private insurance vs. 6.2% Medicare). Patients insured by Medicare (Odds Ratio [OR] = 1.23, 95% Confidence Interval [CI] 1.17–1.29, P < 0.05) and Medicaid (OR = 1.58, 95% CI 1.44–1.73, P < 0.05) had higher likelihoods of being readmitted up to 30 days postoperatively than patients with private insurance. Similarly, patients insured by Medicare (OR = 1.20, 95% CI 1.16–1.25, P < 0.05) and Medicaid (OR 1.52, 95% CI 1.42–1.62, P < 0.05) also had higher likelihoods of being readmitted up to 90 days postoperatively than patients with private insurance. Patients living in geographic areas with the highest median household income values of their state were less likely to be readmitted 30 and 90 days postoperatively than those living in the poorest income quartiles of their state (30-day OR = 0.89, 95% CI 0.85–0.94, P < 0.05 and 90-day OR = 0.91, 95% CI 0.87–0.94, P < 0.05). Blacks were more likely than Whites to be admitted up to 30 and 90 days postoperatively (30-day OR = 1.20, 95% CI 1.11–1.29, P < 0.05 and 90-day OR = 1.08, 95% CI 1.02–1.14, P < 0.05). |
| Wilson S, Marx RG, Pan TJ, et al | Retrospective cohort study (3) | The aim of this study was to identify a set of meaningful hospital and surgeon total knee arthroplasty volume thresholds. | The New York State Department of Health’s Statewide Planning and Research Cooperative System (SPARCS) is a comprehensive data reporting system that collects patient-level detail on all discharges from nonfederal acute-care hospitals. The data set included patient-specific data from 1997 through 2011, as patient identifiers were not available before then. | Using 289,976 patients undergoing primary total knee arthroplasty from an administrative database, authors applied stratum-specific likelihood ratio (SSLR) analysis of a receiver operating characteristic (ROC) curve to generate sets of volume thresholds most predictive of adverse outcomes. The outcomes considered for surgeon volume included 90-day complication and 2-year revision. For hospital volume, we considered 90-day complications and 90-day mortality. | SSLR analysis of the ROC curves for 90-day complication and 2-year revision rates by surgeon volume identified four volume categories: 0 to 12, 13 to 59, 60 to 145, and ≥146 total knee arthroplasties per year. Complication rates decreased significantly (p < 0.05) in progressively higher-volume categories. Revision rates followed a similar pattern but did not decrease between surgeons performing 60 to 145 arthroplasties per year and those performing ≥146 arthroplasties per year. SSLR analysis of 90-day complication and 90-day mortality rates by hospital volume also identified four volume categories: 0 to 89, 90 to 235, 236 to 644, and ≥645 total knee arthroplasties per year. Complication rates decreased significantly (p < 0.05) in progressively higher-volume categories, but the rates did not decrease between hospitals performing 236 to 644 arthroplasties per year and those performing ≥645 arthroplasties per year. Mortality rates for hospitals with ≥645 total knee arthroplasties per year were significantly lower (p < 0.05) than those below the threshold. |
| Xu HF, White RS, Sastow DL, et al | Retrospective cohort study (3) | The purpose of this investigation was to determine if primary payer status is a predictor of increased perioperative risks and postoperative outcomes after total hip replacement (THR) surgery. | A total of 295,572 adult patients who had undergone THR surgery in California, Florida or New York were included. 164,927 (55.8%) of patients had Medicare, 10,170 (3.4%) had Medicaid, 110,150 (37.3%) had private insurance and 2302 (0.8%) were uninsured. 243,193 (82.3%) of all patients were white, 17,028 (5.8%) were black and 16,313 (5.5%) were Hispanic. 166,785 (56.4%) of all patients were female. | The authors analyzed the California, Florida and New York State Inpatient Databases (SID), Healthcare Cost and Utilization Project, Agency for Healthcare Research and Quality to identify patients who underwent THR from 2007 to 2011. Inclusion criteria included patients greater than 18 years of age. Exclusion criteria included patients who had missing insurance information. Demographic characteristics, comorbidities, rates of in-hospital mortality, length of stay (LOS), 30 and 90-day readmission rates were compared for all patients by insurance type. Continuous variables were compared using analysis of variance (ANOVA) and categorical variables were compared using Chi squared test or Fischer's exact test. Logistic regression was used to examine the effect of insurance type on postoperative outcomes. | Medicaid patients incurred a 125% increase in the odds of in-hospital mortality compared to those with Private Insurance (Odds Ratio [OR] = 2.25, 99% Confidence Interval [CI] 1.01–5.01, P < 0.01). Medicaid payer status was associated with the highest statistically significant adjusted odds of mortality or having any complication (OR = 1.26, 99% CI 1.11-1.43, P < 0.001), cardiovascular complications (OR = 1.37. 99% CI 1.04-1.81, P < 0.005), and infectious complications (OR = 1.66, 99% CI 1.35-2.05, P < 0.001) when compared with Private Insurance. Medicaid patients had the highest statistically significant odds of 30-day (OR = 1.63, 99% CI 1.45-1.83, P < 0.001) and 90-day readmission (OR = 1.58, 99% CI 1.44-1.73, P < 0.001). Medicaid payer status was associated with the longest adjusted length of stay (OR = 1.18, 99% CI 1.18-1.19, P < 0.005). |
| Yayac MF, Harrer SL, Janiec DA, et al | Retrospective cohort study (3) | The purpose of this study was to determine whether 90-day episode-of-care (EOC) costs and outcomes were different for patients with Medicare Advantage (MA) plans undergoing total joint arthroplasty compared with traditional Medicare patients | When comparing demographics of the two groups, significant differences were found in age and race (P < 0.0001). Patients enrolled in MA were older on average (74.19 ± 7.11 years) than those enrolled in TM (72.16 ± 6.86 years). No significant differences were found between groups in sex or body mass index, but TM patients significantly differed from MA patients for several comorbidities, including hypertension (40.9% versus 33.8%, P < 0.0001), history of cancer (11.9% versus 8.0%, P < 0.0002), and diabetes mellitus (10.9% versus 7.1%, P < 0.0001) | Authors reviewed claims data for a consecutive series of patients undergoing primary total hip and knee arthroplasty from 2015 to 2018 at their institution with traditional Medicare coverage or MA through a single private insurer. Demographics, comorbidities, 90-day costs, readmissions, complications, and discharge disposition were compared between the groups. A multivariate regression analysis was performed to determine the independent effect of insurance status on EOC costs and outcomes | Of the 10,869 patients in the study, 1,076 (9.9%) were covered under an MA plan. MA patients were more likely to be discharged to a rehabilitation facility (19% versus 14%, P < 0.0001). No significant differences were observed in length of stay (1.88 versus 1.88 days, P = 0.1439), complications (3.9% versus 3.5%, P = 0.4554), or readmissions (5.9% versus 4.9%, P = 0.1893). EOC costs were significantly higher for the MA group ($21,347 versus $19,551, P < 0.0001) |
